# An investigation into the role of chronic *Schistosoma mansoni* infection on Human Papillomavirus (HPV) vaccine induced protective responses

**DOI:** 10.1371/journal.pntd.0007704

**Published:** 2019-08-26

**Authors:** Vicky Gent, Rebecca Waihenya, Lucy Kamau, Ruth Nyakundi, Peris Ambala, Thomas Kariuki, Lucy Ochola

**Affiliations:** 1 Department of Zoology, Jomo Kenyatta University of Agriculture and Technology, Nairobi, Kenya; 2 Department of Tropical and Infectious Diseases, Institute of Primate Research (IPR), Karen, Kenya; 3 Alliance for Accelerating Excellence in Science in Africa (AESA), Karen, Kenya; University of Manchester, UNITED KINGDOM

## Abstract

**Background:**

*Schistosoma mansoni* is one of the most common helminth infections affecting a large population of people in sub-Saharan Africa. This helminth infection is known to cause immunomodulation which has affected the efficacy of a number of vaccines. This study examined whether a chronic schistosoma infection has an effect on the immunogenicity of HPV vaccine which is currently administered to girls and women aged 9 to 24. Little is known about the immune responses of the HPV vaccine in individuals with chronic schistosomiasis.

**Methods:**

This study was carried out at the Institute of Primate Research (IPR) and involved an Olive baboon model. The experimental animals were randomly placed into three groups (n = 3–4); Two groups were infected with *S*. *mansoni* cercaria, and allowed to reach chronic stage (week 12 onwards), at week 13 and 14 post-infection, one group was treated with 80mg/kg of praziquantel (PZQ). Sixty four weeks post schistosoma infection, all groups received 2 doses of the *Cervarix* HPV vaccine a month apart. Specific immune responses to the HPV and parasite specific antigens were evaluated.

**Results:**

Animals with chronic *S*. *mansoni* infection elicited significantly reduced levels of HPV specific IgG antibodies 8 weeks after vaccination compared the PZQ treated and uninfected groups. There was no significant difference in cellular proliferation nor IL-4 and IFN-γ production in all groups.

**Conclusion:**

Chronic *S*. *mansoni* infection results in reduction of protective HPV specific IgG antibodies in a Nonhuman Primate model, suggesting a compromised effect of the vaccine. Treatment of schistosomiasis infection with PZQ prior to HPV vaccination, however, reversed this effect supporting anti-helminthic treatment before vaccination.

## Author summary

In sub-Saharan Africa countries, vaccines are administered to people who may suffer from existing infections, especially helminth infections. These infections are known to modulate immune responses rendering some vaccines ineffective. The impact of helminth infections such as schistosomiasis on a recently introduced Human Papillomavirus (HPV) vaccine on infected or treated populations and the degree or duration has not been clearly elucidated. This study was set up to investigate whether a chronic schistosoma infection compromises the specific immune responses elicited by the HPV vaccine.

## Introduction

Human Papillomavirus (HPV) remains one of the most common sexually transmitted viruses in the world and is responsible for cervical cancer. Cervical cancer has been categorized as the 3^rd^ most common cancer affecting women in the world. It has been estimated that 527,624 women are diagnosed with cervical cancer each year and 266,672 die due to the complications caused by the disease worldwide. In Africa, the incidence of cervical cancer is high, approximately 99,038 cases were recorded in 2012 [1]. The burden of cervical cancer in Sub-Saharan Africa has been steadily increasing and this had led to the introduction and testing of HPV vaccines in Africa [2–6]. Currently, three licensed vaccines against HPV are available; the quadrivalent vaccine *Gardasil* which provides protection against HPV 6,11,16, 18, bivalent vaccine *Cervarix* which confers protection against the 2 variants, HPV 16 and HPV 18 [7] and a nanovalent vaccine *Gardasil9* which protects against HPV 16, 18, 31, 33, 45, 52 and 58 subtypes [8,9]. These vaccines contain virus-like-particles (VLPs) consisting of the L1 capsid protein. These proteins are highly immunogenic resulting in high levels of serum antibody immune responses once injected intramuscularly [10]. This results in high levels of efficacy for protection, however no immune correlates have been identified for HPV vaccination [11]. A number of trials have been conducted to document levels of efficacy associated with persistent levels of IgG and IgA antibodies [12] as well as the prevention of high grade Cervical Intraepithelial Neoplasia (CIN) and cervical cancer [8–11,13]. HPV vaccination programmes are underway in several countries, with Kenya expected to roll out free HPV vaccines in 2019 [14].

It has been suggested that a chronic helminth infections (including schistosomiasis) during the time of vaccination might impair the induction of protective immune responses elicited by vaccines. The ability of helminths to modulate the host’s immune responses ensures its own survival. Immune modulation has been considered to have a “spill over” effect and reduce immune responses to other antigens [15] contributing to lowered vaccine responses observed in developing countries where helminth infections are endemic [15,16,17].

Schistosomiasis, has been estimated to affect more than 250 million people with majority of cases occurring in Sub-Saharan Africa [18]. Several studies have shown that parasitic infections, especially schistosomiasis, impair long-term responses of certain vaccines such as BCG [19], TB [20], hepatitis B [21] and tetanus toxoid [22] that require a predominant Th1 response to be effective. During a chronic helminth infection, there is a characteristic induction of a Th2 response which down regulates Th1 responses. Indeed during a schitosome infection, Th1/Th2 dichotomy that occurs is attributed to the Th2 immune response (especially the production of IL-4), IL-10 and T regulatory (Tregs) cells response. The Tregs are shown to downregulate the Th1 responses as well as limit the Th2 responses. These immune response is essential for host’s survival against the toxic effects of the Schistosoma egg antigens [23–25].[20,22].

Studies have shown that early anti-parasite treatment can prevent immunomodulation caused by these parasite antigens thereby improving vaccine efficacy [26]. For instance, it has been reported that elimination of *S*. *mansoni* by praziquantel treatment in mice prior to receiving the HIV-1 C vaccine resulted in restored T cell responses and significantly increased IFN-γ produced by ConA- stimulated splenocytes compared to untreated mice. It was indicated that restoration of HIV-1C vaccine specific T cell responses after antihelminthic treatment was time dependent [27]. Therefore, from these previous studies it can be construed that antihelminthic treatments leading to the elimination or reduction of the helminth infection reverses the Th2 response to Th1 response. A strong Th1 immune response is necessary for effective vaccine induced immune responses.

A number of studies have assessed the safety and efficacy of the HPV vaccine in the community. While a few studies have considered how immune-modulating infections, such as malaria and helminths, could influence the potency of the HPV vaccine. Recent studies have evaluated the immunogenicity of HPV vaccine in helminth exposed females in Uganda and Tanzania [28,29].These studies found no significant difference between antibody levels in helminth infected and uninfected individuals after receiving the HPV vaccine. However, low sensitivity of Kato Katz in the detection of eggs, the duration of exposure and intensity of the helminth infection prior to vaccination, were not evaluated and these could influence antibody levels.

There is a need, therefore, to evaluate how the degree of helminth infection influences the efficacy of HPV vaccine. Baboons (*Papio anubis*) are natural hosts of *Schistosoma mansoni*. The pathology and immune responses to chronic *S*. *mansoni* in baboons mimic those observed in humans. This study was therefore designed with a goal to investigate the effect of a chronic *S*. *mansoni* infection on the immunogenicity of the HPV bivalent vaccine using a baboon model.

## Materials and methods

### Ethics statement

This study and all experimental protocols were approved by the Institutional Science and Ethics Committee (ISERC) of the IPR, Karen, Nairobi, Kenya (study IRC/08/10), whose membership is constituted based on guidelines issued by the World Health Organization for committees that review biomedical research, by the NIH, by PVEN, and by the Helsinki Convention on the Humane Treatment of Animals for Scientific Purposes. The IRC-ACUC is nationally registered by the National Commission for Science, Technology, and Innovation, Kenya.

### Study design

This study involved a total of ten Olive baboons (*Papio anubis*) which were housed at the Institute of Primate Research (IPR [www.primateresearch.org]), Karen, Nairobi, Kenya. This was according to institutional standards and guidelines for primate welfare and housing based on the *International Guiding principles for Biomedical research Involving Animals development* by the Council of International Organizations of Medical Sciences in 1985: Appendix A of the European *Convention for the Protection of Vertebrate Animals Used for Experimental and Scientific Purposes* (ETS 123,2006), *Convention for International Trade in Endangered Species*, *the U*.*S*. *Guide for the Care and Use of Laboratory Animals* and the European Primate Resources Network/Primate Vaccine Evaluation Network [PVEN]. These guidelines were also in compliance with the Association for Assessment and Accreditation for laboratory Animal Centers; and the *Statement of Compliance with standards for Humane Care and use of Laboratory animals by Foreign Institutions* issued by the National Institutes of Health (NIH)[30].

The baboons were housed in outdoor group cages to allow social interactions and only moved to individual cages during stool and blood sample collection. Both grouped and individual cages were designed to allow natural light and dark cycles and they had *ad libitum* access to water. They were fed daily with monkey cubes (Unga Farm Care, Ltd, Nairobi, Kenya) and supplemented with fruits and vegetables [30].

Ten Male and female, sub-adult, Olive baboons weighing approximately 5 to 9Kgs were selected for the study. The animals first underwent screening to ensure that they were free from ectoparasites, protozoan and helminth infections, tuberculosis and Simian Immunodeficiency virus (SIV).

**Fig 1:**
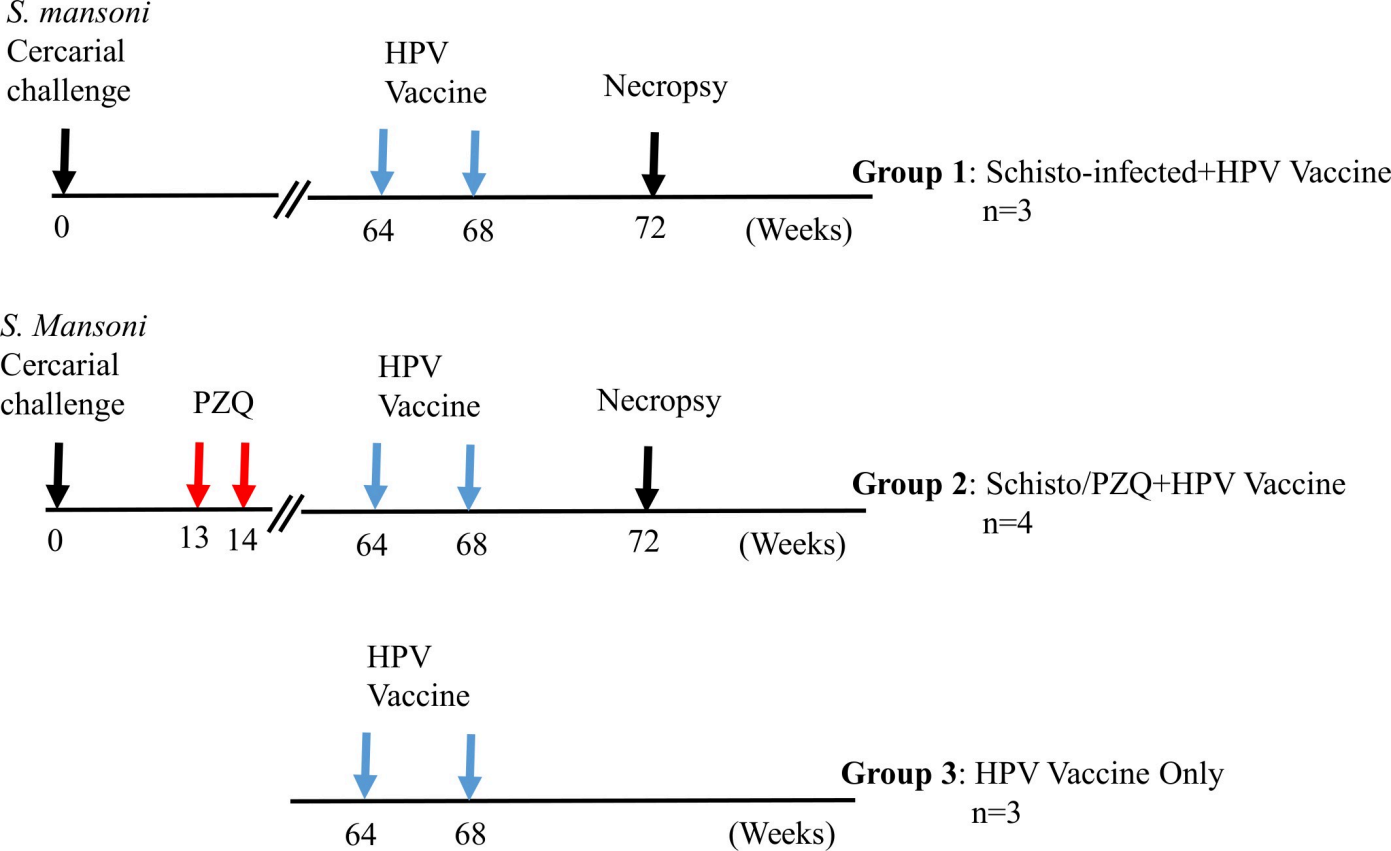
Experimental design. Three groups of animals were included in the experiment. i) animals infected with S. mansoni cercariae and later received *Cervarix* HPV vaccine (Schisto infected+HPV), ii) animal infected with S. mansoni, treated with praziquantel (PZQ) before receiving the HPV vaccine (Schisto/PZQ+HPV) and iii) animals that received the HPV vaccine only.

The baboons were randomly placed in 3 groups. Two groups, Schisto-infected+HPV; (n = 3) and Schisto/PZQ+HP; (n = 4)were infected percutaneously with 500 *S*. *mansoni cercaria* as previously described [31]. At week 13 and 14, after infection, the Schisto/PZQ+HPV group was treated twice with Praziquantel (PZQ; Balcitricide, Bayer Schering Pharma) at a dose of 80mg/kg, via oral intubation, to clear the Schistosoma infection. At week 64 post infection, the two groups exposed to *S*. *mansoni* infection, Schisto-infected+HPV, Schisto/PZQ+HPV group, and an additional HPV vaccine only group (HPV-only n = 3) received 0.5ml *Cervarix* HPV vaccine containing a dose of 20μgHPV16 and20μgHPV18. The vaccine was administered via Intramuscular injection at the *Vastas lateralis* muscle of the right hind leg. The animals were monitored for 15 to 60 minutes for any side effects, such as reddening and swelling, at the point of vaccination. As it has been observed that 2 doses of the HPV bivalent vaccine is just as effective as 3 doses [7,32] the animals received a vaccine boost (the same dosage) four weeks later. (Fig 1).

**Fig 2:**
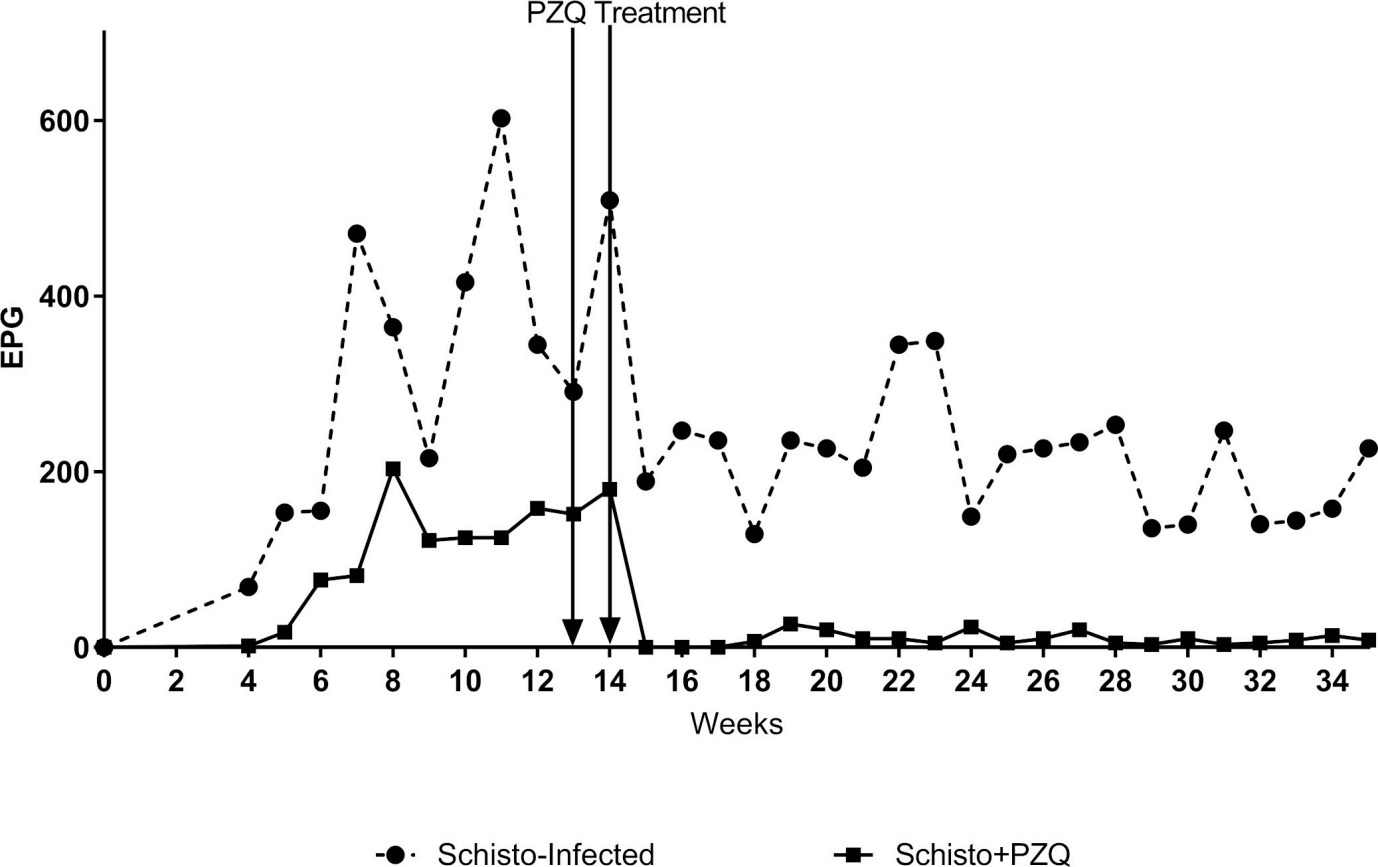
Egg burden in *S*. *mansoni* infected baboons. Egg burden was monitored weekly over a 35 week period and egg counts were done using the Katokatz method. Egg quantity was calculated as number of eggs per gram (EPG) of faeces.

Schistosome infection in the Schisto-infected and Schisto/PZQ groups was monitored weekly (beginning week 4 post cercarial challenge) by examination of parasite eggs in the animals faeces using the Kato Katz method. By the time of vaccination, animals with an active schistosome infection (group) had an average of 179±74.9eggs/grams of faeces while those that received PZQ treatment (group) displayed a significant reduction, approximately 95.4% (26 ±6.675eggs/grams of faeces) (P<0.001), at week 15 post cercarial challenge. This ensured elimination of the schistosome infection in the Schisto/PZQ group prior to HPV vaccination (Fig 2).

**Fig 3:**
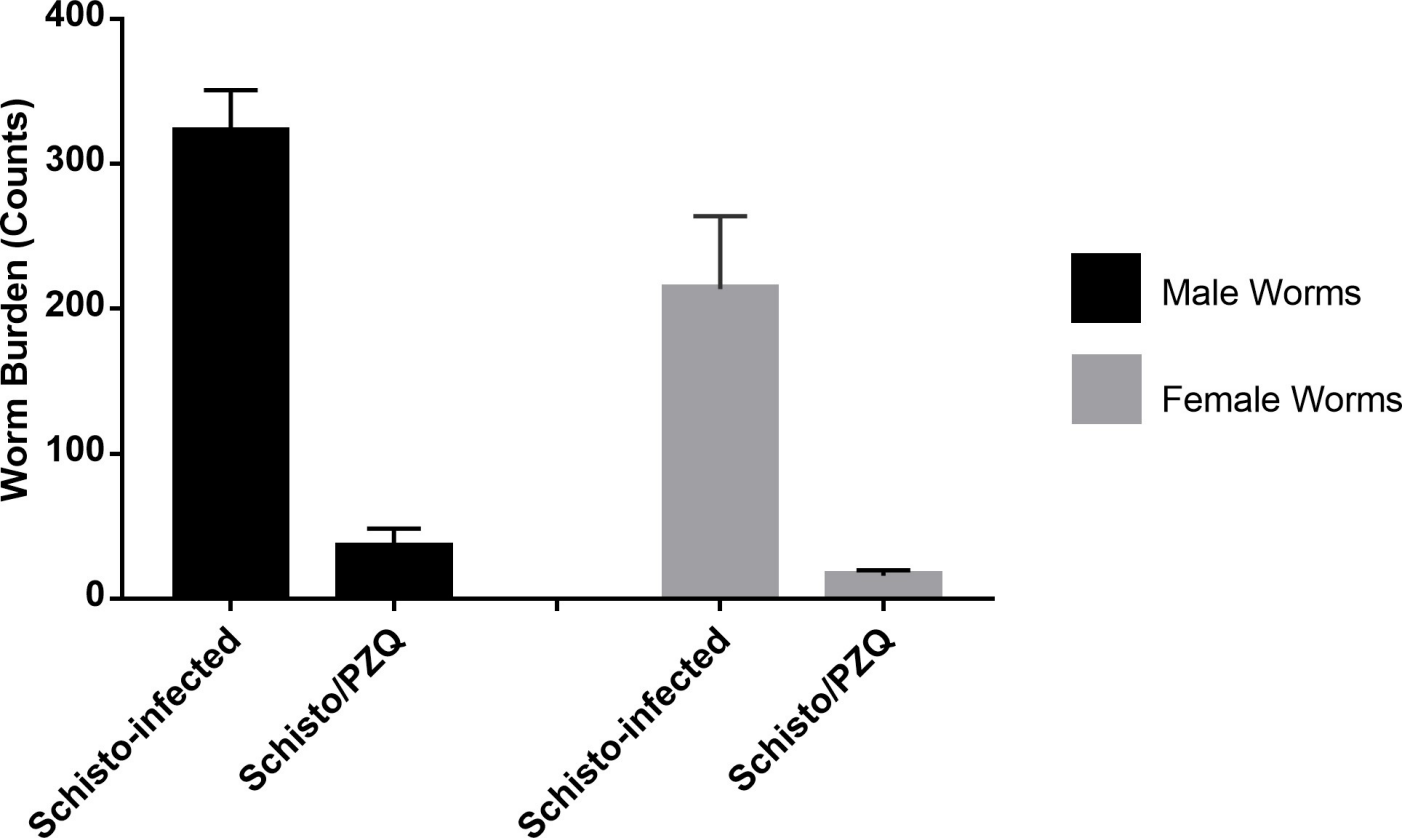
Adult Schistosoma worms recovered from the experimental animals. Total male worm counts, indicated by the black bar, and female worm counts, indicated by the grey bar, from the Schisto-infected and Schisto/PZQ animal groups.

The animals were euthanized by intramuscular administration of Ketamine-HCL at a dose of 10mg/kg and Xylazine at a dose of 0.5mg/ml which sedated the animals followed by intravenous administration of 100mg/kg of Sodium pentobarbitone (Eutha-Naze; Bayer HealthCare). Perfusion of the worms from the mesenteric vasculature and liver by the administration of citrated saline through the abdominal aorta was done as previously described [33]. The recovered male and female worms were counted and recorded as shown in Fig 3. Throughout the experiment, blood was collected prior to the first and second vaccinations, and two other time points 2 weeks apart. Blood samples underwent immunological responses evaluation.

### Antibody assay

Levels of HPV-specific IgG antibodies present in sera were determined using Enzyme Linked Immunosorbent Assay (ELISA). Briefly, flat bottomed 96-well ELISA plates were coated with 1.25μg HPV16/18 diluted in 1X Phosphate Buffered Saline (PBS) at 50μl per well then incubated at 4°C overnight. The plates were washed with 0.05% PBS-Tween20 (wash buffer), blocked with 3% BSA in wash buffer and incubated at 37°C for 1 hour. The plates were washed 5 times and 50μl of serum samples diluted 1:200 in wash buffer was added in duplicate. For the blanks wells 50μl of wash buffer was included. This was followed by incubation at 37°C for 2 hours. The plates were washed five times and 50μl anti-monkey secondary antibody (Sigma-Aldrich, Dorset, UK), diluted 1:2000 in wash buffer, was added per well and incubated for 1hour at 37°C. 50ul of 3,3,5,5-tetramethylbenzidine (TMB, Kirkegaard & Perry Labs, USA) was added per well and the color was allowed to develop for 30 minutes The plates were read using the ELISA reader (Biotek Elx808) at a wavelength of 630nm.

### Peripheral Blood Mononuclear Cell (PBMC) culture

Whole blood was mixed in a dilution of 1:2 blood to Alsevers solution (0.055% citric acid, 0.42% sodium chloride, 0.8% trisodium citrate, 2.05% dextrose) followed by layering on Ficoll paque (Pharmacia Ciotech, St. Albans, UK). The blood was centrifuged at 2000rpms for 20 minutes at 24°C. The buffy coat containing the Peripherial blood mononuclear cells was collected and washed twice with 1X PBS at 1500rpm for 10 minutes at 4°C. The isolated PBMCs were cultures to determine antigen specific T cell reactivity and cytokine production as previously described [30]. Under sterile conditions, PBMCs (1 X 10^6^ cells) were plated in 48-well culture plates in 1 ml of complete culture media (RPMI-1640; Sigma-Aldrich, St. Louis, MO, 10%FBS; Gibco, Canada, 1%HEPES; Sigma-Aldrich, Dorset, UK, 1% Gentamycin; Sigma-Aldrich, Dorset, UK, 1% L-glutamin; Fisher Biotech, Wembley, Australia). 5μg/ml Schistosoma Egg Antigen (SEA; Schistosome Biological Supply Center, Theodor Bilharz Research Institute, Egypt) and 5μg/ml Soluble Worm Antigen Preparation (SWAP; Schistosome Biological Supply Center, Theodor Bilharz Research Institute, Egypt) and HPV Ag (Glaxosmithkline, UK) at a final concentration of 1.25μg/ml were added to the test wells. 5μg/ml of Concanavalin A (CONA; Sigma-Aldrich, Dorset, UK) was included as positive control while medium only (no stimulant) cells acted as negative (background) control. The cultures were incubated at 37°C 5% CO_2_. Culture supernatant was collected at 48hours and 72hours and stored at -45°C.

### Proliferation assay

Proliferation of PBMCs was performed as described previously (34, 35). 1 X10^6^ cells were stained with 5mM of 5(6)- Carboxyfluorescein diacetate N-Succinimidyl ester (CFSE; Invitrogen, Germany) and cultured in the presence of antigens and mitogens as mentioned in the PBMC cultures[34,35]. After 72 hours of incubation, cells were harvested and stained with 5μl of 7-Aminoactinomycin D (7AAD, Sigma-Aldrich, Dorset, UK). Samples were run on the FACS caliber (4 colour) cytometer (BD, biosciences, USA) and data acquired using the Cell Quest Pro program. Analysis was done using Flowjo software Version 10(Ashland, OR) with the gating strategy shown in supplementary 1 (S1 Fig).

### Cytokine assay

Antigen-specific IL-4 and IFN-γ were detected from culture supernatants at 48 and 72 hours respectively using Human IL-4 ELISA (Mabtech, Sweden) and Monkey IFN-γ ELISA (Ucytech, Netherlands) kits, as indicated by the manufactures instructions and as previously described [30]. Briefly, the supernatants were thawed and diluted with Dilution buffer (PBS 0.5% (w/v) BSA 0.05% (w/v) Tween-20) at a ratio of 1:1. One hundred microliters of the coating antibody was added to the wells of the ELISA plate (Nunc MaxiSorp) and incubated overnight at 4°C. The plates were washed twice with PBS and 200μl blocking buffer (PBS 0.05% Tween 20 1% BSA) was added after which the plate was incubated for 1 hour at room temperature. The plates were washed 5 times with wash buffer (PBS 0.05%Tween 20). One hundred microliters of the diluted samples and standards were plated in duplicate, except for the blanks, the plates were incubated at room temperature for 2 hours. After incubation, the plates were washed 5 times with the wash buffer, then 100μl of biotinylated secondary antibody was added to each well and left to incubate for 1 hour at room temperature. After washing, 100μl of diluted streptavidin-HRP was added to each well and left to incubate at room temperature for 1 hour. The plate was washed and 100μl of TMB substrate (Kirkegaard & Perry Labs, USA) was added to each well. The plate was placed in the dark for 25minutes to allow the chromogenic substrate to develop a blue colour. The plates were read using the ELISA reader (Biotek Elx808) at a wavelength of 630nm. Cytokine concentration was reported as picograms (pg) and extrapolated from the standard curve using Graphpad prism V.7 software. The mean of the blanks (Background) were subtracted from the response Optical densities.

### Statistical methods

Antibody and proliferation data was normalized and analyzed using one-way ANOVA, followed by Tukey post hoc test for multiple comparison. Cytokine data on the other hand was analyzed using Kruskal Wallis, followed by Dunn’s post hoc analysis. Egg counts derived from Katokatz was analyzed using Wilcoxon matched-pairs sign rank test. All analysis were done using the GraphPad Prism software Version 7.00 for Windows (GraphPad Software, La Jolla California USA, www.graphpad.com).Statistical level of significance was set at p<0.05.

## Results

### Chronic *S*. *mansoni* infection lowers HPV specific IgG response

**Fig 4:**
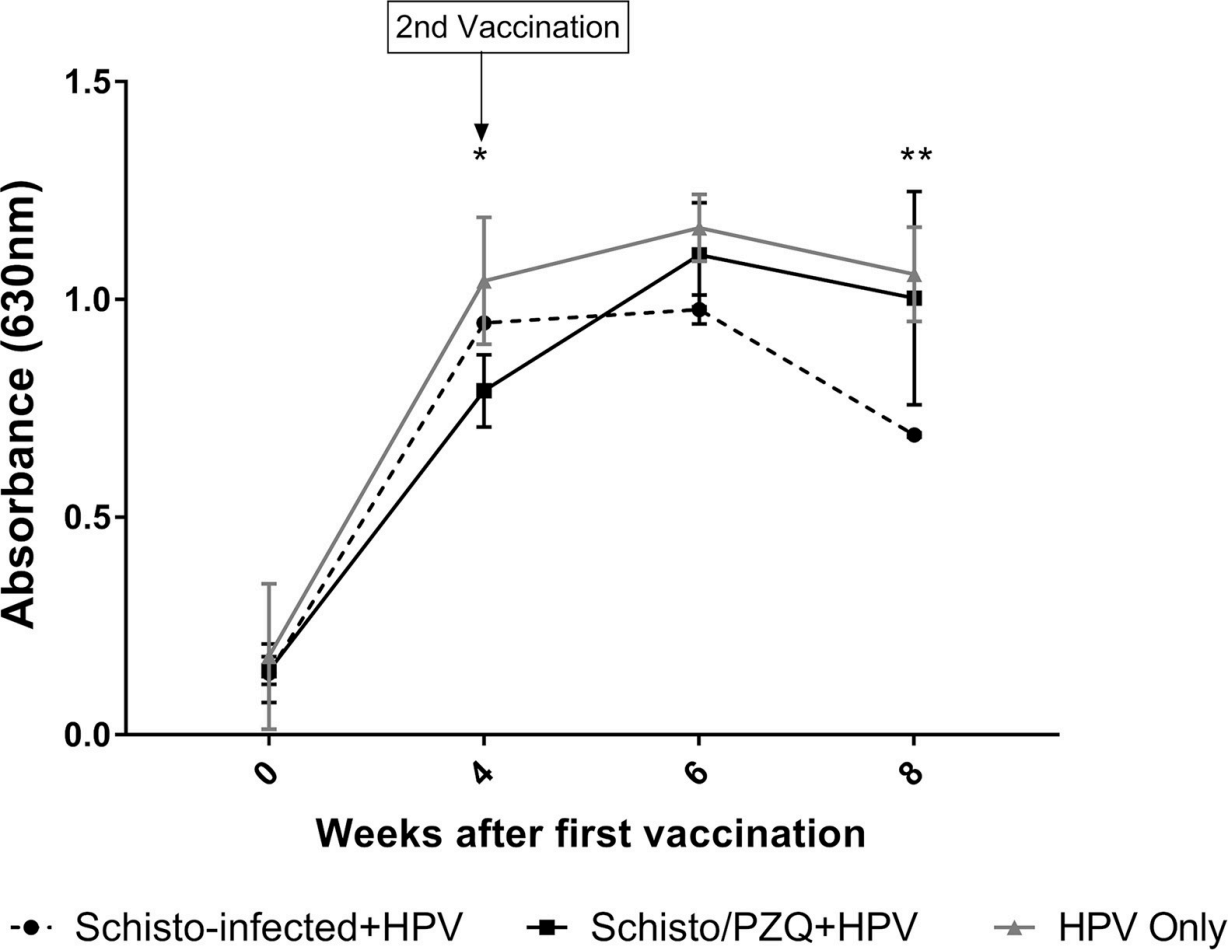
HPV specific IgG antibody responses in Optical densities for Group 1 Schisto.-infected+HPV, Group 2 Schisto/PZQ+HPV and Group 3 HPV-only. Week 0 (pre-vaccination), Week 4 (4 weeks after the 1^st^ vaccination), Week 6 (6 weeks after the 1^st^ vaccination and 2 weeks after the 2^nd^ dose), Week 8 (8 weeks after 1^st^ dose and 4 weeks after 2^nd^ dose). The experimental points represent the mean IgG OD of each group at each time point. Groups are compared to the control group (Group 3 HPV-Only). Values were considered statistically significant when p≤0.05 and assigned *p = 0.01–0.05 (group 2 vs group3), **p = 0.001 (group 1 vs group3).

At the 64^th^ week the animals were vaccinated with the 1^st^ dose of HPV vaccine. They received the 2^nd^ dose of the vaccine 4 weeks later. The levels of HPV specific IgG antibodies in serum were assessed by ELISA. (Fig 4).

There was a general increase in HPV specific serum IgG antibodies produced during this study, with the lowest levels observed prior to HPV vaccination. After administration of the first dose of the vaccine, Schisto-infected+HPV group, Schisto/PZQ+HPV group and HPV-only group showed an increase (6.7 fold, 5.3 fold and 5.8 fold respectively) in optical density (OD) which corresponds to levels of HPV specific serum IgG antibodies present in the samples. Four weeks after the first dose of vaccine was administered, the HPV-only group had a significantly higher OD levels (1.042±0.084) compared to the Schisto/PZQ+HPV group (0.946±0.007) (p = 0.0306). The HPV specific IgG levels of animals of the HPV-only and Schisto/PZQ groups continued to increase 2 weeks after the 2nd vaccine dose was given. While these levels declined significantly (p = 0.0301) between 6^th^ and 8^th^ week after the first vaccination in the Schisto-infected animals. At the 8^th^ week, HPV-vaccine only and Schstio/PZQ groups had significantly higher OD levels compared to Schisto-infected+HPV (p = 0.0015 and p = 0.0066 respectively). However, at this same time point, there was no significant difference between HPV-only and Schisto/PZQ+HPV (p = 0.8259). This indicates that HPV specific IgG antibody response is compromised during a chronic *S*. *mansoni* infection. (Fig 4).

### Chronic *S*. *mansoni* infection has no significant effect on lymphoproliferation response

Histograms (S1 Table) were analyzed using Flowjo V.10 proliferation modelling to fit the raw data. This proliferation modelling provided the average number of cells that lost any intensity of CFSE (CFSE^low^), specifically, the number of cells in subsequent generations. The CFSE^low^ of the 3 groups over the 8 weeks were compared.

**Fig 5:**
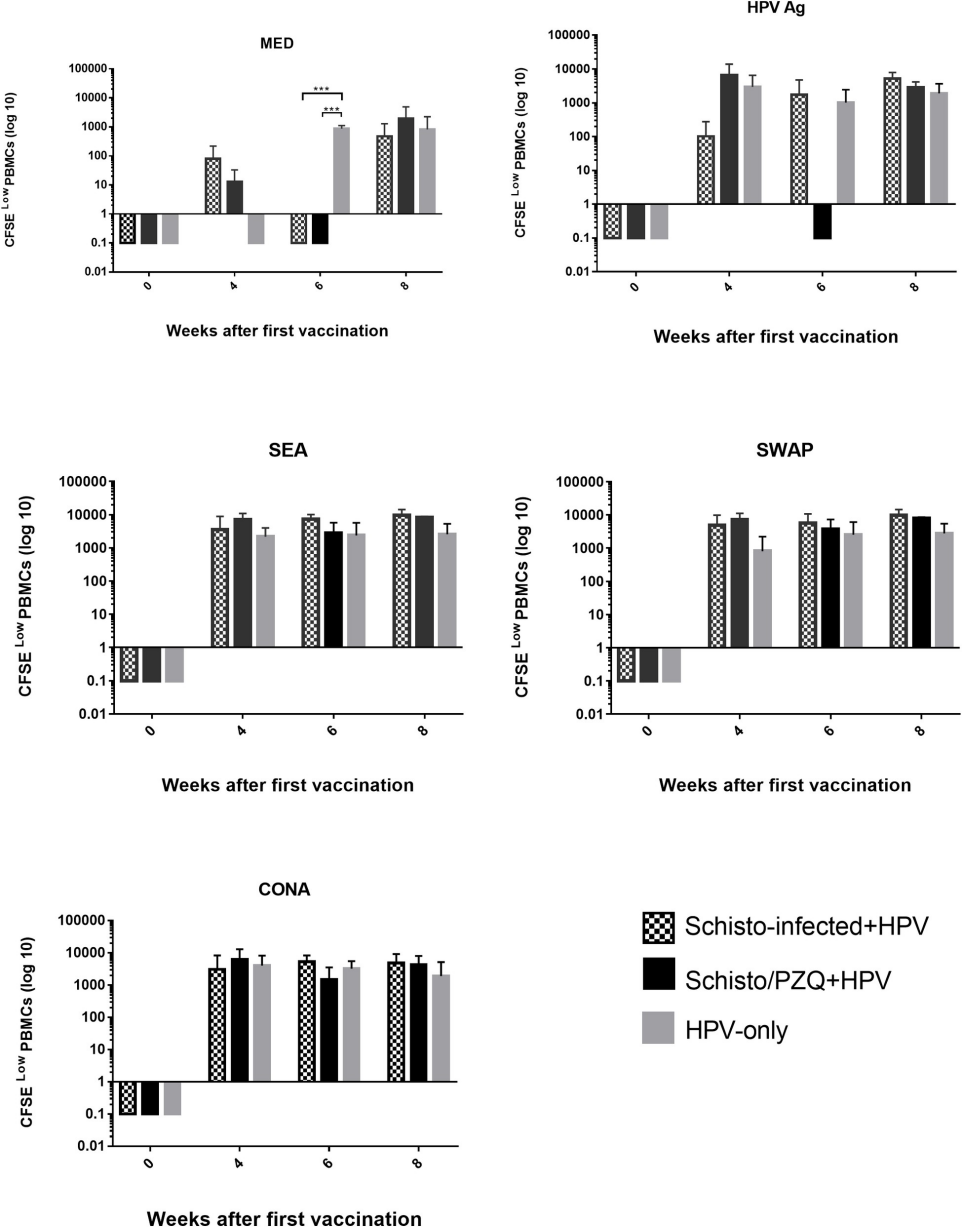
CFSE^low^ PBMCs. Illustrates the average number of cells that have lost any level of CFSE labelling after 72 hours in culture during specific (HPV, SEA and SWAP), non-specific stimulation (CONA) and absence of stimulant (MED) in Schisto-infected+HPV, Schisto/PZQ+HPV and HPV-only (Control) groups. PBMCs were collected from the 3 groups at 4 time points; Week 0 (pre-vaccination), Week 4 (4 weeks after the 1^st^ vaccination and before the booster), Week 6 (6 weeks after the 1^st^ vaccination and 2 weeks after the 2^nd^ dose), Week 8 (8 weeks after 1^st^ dose and 4 weeks after 2^nd^ dose). The bars represent the mean number of CFSElow PBMCs of each group at each time point. Data was normalized according to the baseline results (Week 0) and analyzed using one-way ANOVA followed by Dunn multiple comparison test. Data was considered statistically significant if p≤0.05 and assign *** p = 0.0006.

PBMCs that were cultured in the absence of any stimulant (MED) showed the least number of cells that lost CFSE fluorescence intensity, thus indicating that very low cellular division occurred in these cells as compared to the PBMCs stimulated with HPV Ag, SEA, SWAP and CONA (Fig 5).

The CFSE^low^ levels of PBMCs collected at the 8^th^ week and cultured in the presence of HPV antigen were higher than those collected prior to vaccination for the Schisto-infected+HPV, Schisto/PZQ+HPV and HPV-only groups (p = 0.0589, p = 0.7920, p = 0.7221). PBMCs of Schisto/PZQ+HPV group and HPV-only group collected 4 weeks after the 1^st^ dose of vaccination had higher CFSE^low^ cell numbers compared to those of Schisto-infected+HPV thus indicating a slightly higher proliferation capacity in the Schisto/PZQ+HPV and HPV-only, however, this was not significantly different (p = 0.2818, p = 0.7819). However, PBMCs of the Schisto-infected+HPV collected at the 6^th^ and 8^th^ week had the highest levels of CFSE^low^ cells though this was not significantly different when compared to the other groups.

Slightly lower proliferation was observed in HPV-Only PBMCs when cultured in SEA, while the PBMCs from Schisto-infected+HPV group and Schisto/PZQ+HPV collected at 4^th^, 6^th^ and 8^th^ week had high levels of CFSE^low^ cells. This trend was also observed in PBMCs cultured with SWAP with Schisto-infected+HPV and Schisto/PZQ+HPV having higher levels of CFSE^low^ cells compared with the HPV-only group. Similar to what was observed in PBMCs cultured with HPV Ag, the PBMCs cultured with SEA, SWAP and CONA had higher CFSE^low^ cell levels after the 8 weeks compared with Week 0 (Fig 5).

### Chronic *S*. *mansoni* infection has no significant effect on cytokine response

**Fig 6:**
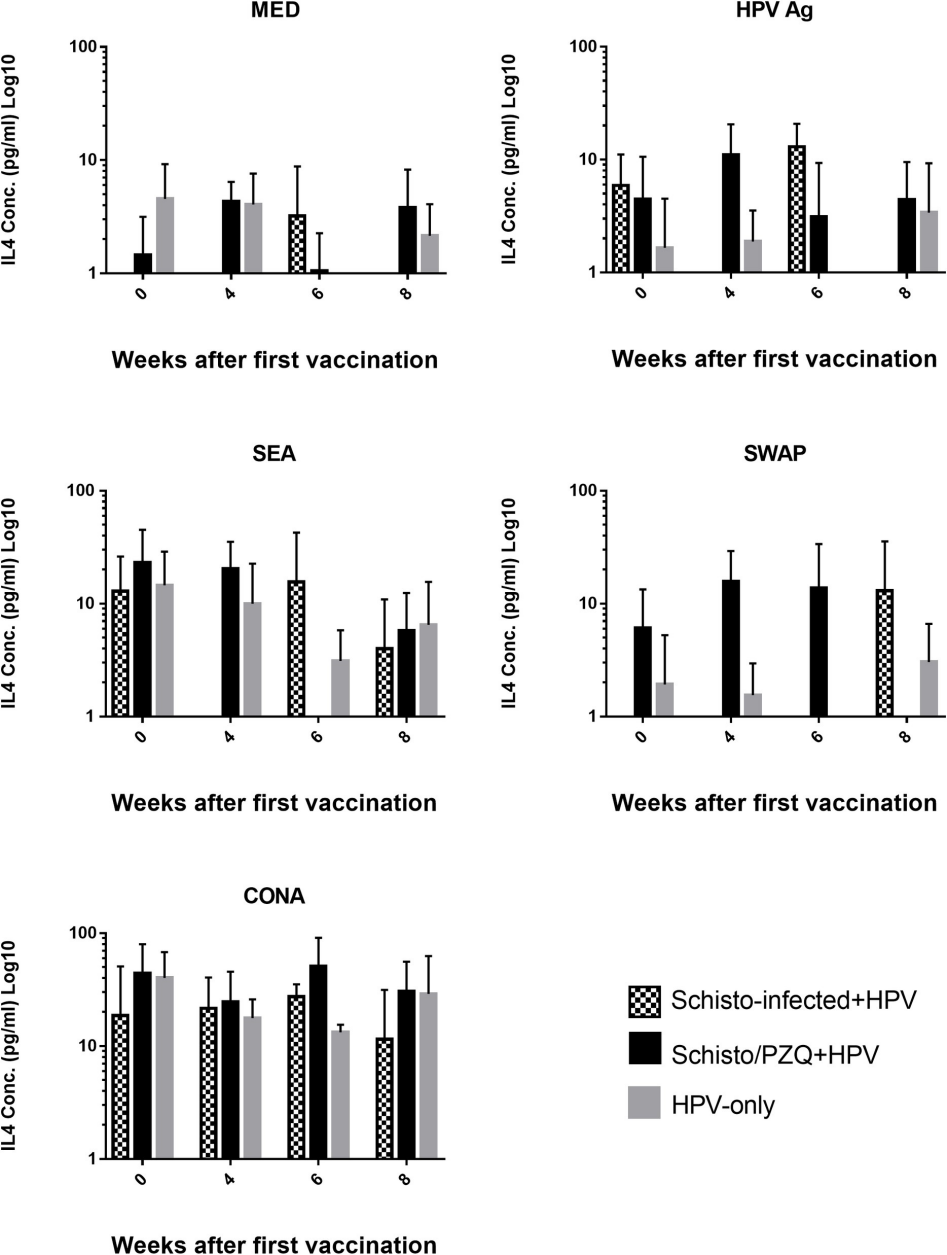
IL-4 cytokine concentrations. Production of IL-4 cytokine in supernatants during Specific (HPV, SEA and SWAP) and non-specific stimulation (CONA) and absence of stimulant (MED) in Schisto-infected+HPV, Schisto/PZQ+HPV vaccine and HPV-only (Control) groups. PBMCs collected from the 3 groups (n = 3) at the 4 time points; Week 0 (pre-vaccination), Week 4 (4 weeks after the 1^st^ vaccination and before the booster), Week 6 (6 weeks after the 1^st^ vaccination and 2 weeks after the 2^nd^ dose), Week 8 (8 weeks after 1^st^ dose and 4 weeks after 2^nd^ dose). These PBMCs were stimulated with respective Antigens (HPV, SEA, SWAP and CONA) for 48h, after which the supernatants were analyzed using ELISA. The bars represent the mean IL-4 levels of each group at each time point. Non-parametric analysis (Kruskal Wallis, Dunn’s post hoc analysis) was applied. Data was considered statistically significant if p≤0.05.

The levels of Th2 cytokine, IL-4, secreted by the PBMCs after non-specific stimulation with ConA, parasite specific stimulation with SEA and SWAP, and HPV antigen specific stimulation was quantified for Schisto-Infected+HPV, Schisto/PZQ+HPV and HPV-only using sandwich ELISA. The responses against the stimulants of Schisto-infected+HPV and Schisto/PZQ+HPV were compared to the HPV-only over the 8 weeks after the 2 doses of the HPV Vaccine was administrated (Fig 6).

Highest levels of IL-4 were observed in PBMCs that underwent non-specific stimulation by CONA while the lowest levels of IL-4 were observed in PBMCs cultured in the absence of stimulant (MED). Stimulation with the HPV Ag generally produced very low levels of IL-4 cytokines in PBMCs collected over the 8 weeks. Prior to vaccination (week 0), Schisto-infected+HPV produced the highest levels of IL-4 (5.89±3.021pg/ml) while the HPV-only group produced the least amount of IL-4 (1.653±1.169pg/ml). HPV-only group continued to produce low levels of IL-4 for the 8 weeks. Four weeks after the 1^st^ dose was administered the PBMCs from Schisto-infected+HPV produced very low levels of IL-4 (below the detectable ELISA threshold), while Schisto/PZQ+HPV showed an increase and produced the highest level (11.01±4.757pg/ml).

Peripheral Blood Mononuclear cells cultured in the presence of SEA illustrated relatively high levels of IL-4 cytokines in the 3 groups prior to vaccination, with the highest observed in Schisto/PZQ+HPV (23.26±10.89pg/ml). Schisto/PZQ+HPV maintained the highest IL-4 until the 6^th^ week when there was a decrease to levels that were below the ELISA threshold. At this time point, Schisto-infected+HPV had the highest level of IL-4. At the 8^th^ week, the 3 groups produced similar levels of IL-4, with HPV-only group having slightly higher levels.

SWAP stimulated PBMCs showed HPV-only group producing very low levels of IL-4 prior to vaccination, and these levels remained negligible until the 8^th^ week. Schisto/PZQ+HPV produced higher levels of IL-4 prior to and after vaccination, while the Schisto-infected+HPV group only showed an increase in IL-4 levels at the 8^th^ week (Fig 6).

**Fig 7:**
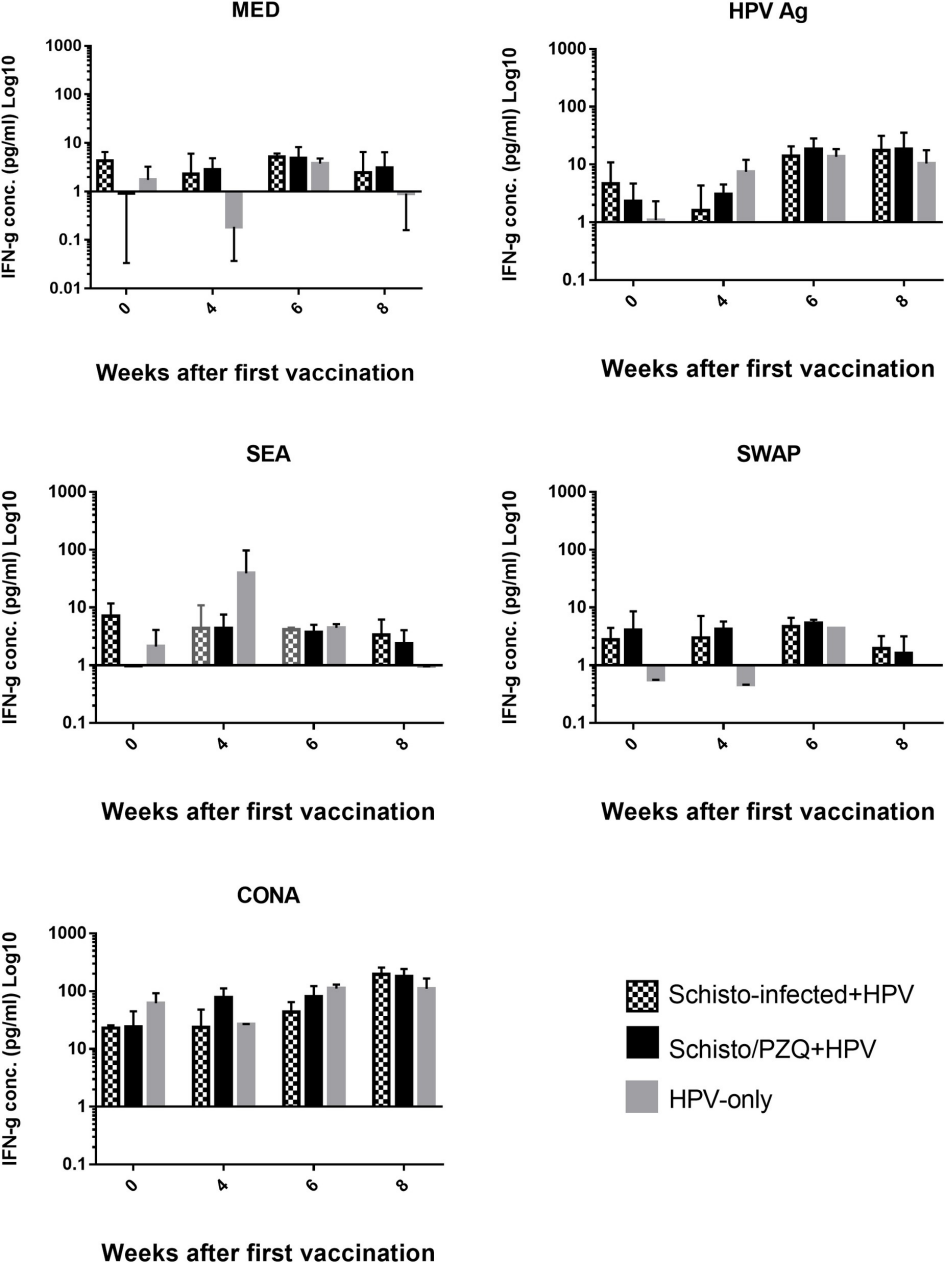
IFN-γ cytokine concentrations. Production of IFN-γ cytokine in supernatants during Specific (HPV, SEA and SWAP) and non-specific stimulation (CONA) and absence of stimulant (MED) in Schisto-infected+HPV, Schisto/PZQ+HPV vaccine and HPV Vaccine only(Control) groups. PBMCs collected from the 3 groups (n = 3) at the 4 time points; Week0 (Pre-vaccination), Week 4 (4 weeks after the 1^st^ vaccination and before the 2^nd^ dose), Week 6 (6 weeks after the 1^st^ dose, 2 weeks after the 2^nd^ dose), Week 8 (8 weeks after the 1^st^ dose and 4 weeks after the 2^nd^ dose) underwent stimulation with respective stimulants for 72hours, after which the supernatants were analyzed using ELISA. The bars represent the mean IFN-γ levels of each group at each time point. Non-parametric analysis (Kruskal Wallis, Dunn’s post hoc analysis) was applied. Data was considered statistically significant if p≤0.05.

The levels of Th1 cytokine, IFN-γ, secreted by the PBMCs after non-specific stimulation with ConA (used as a positive control), Parasite specific stimulation with SEA and SWAP, and HPV antigen specific stimulation was quantified for the Schisto-Infected+HPV, Schisto/PZQ+HPV group and HPV-Only group using sandwich ELISA. Cells that were unstimulated (MED) were used as a negative control. The responses against the stimulants of Schisto-infected+HPV and Schisto/PZQ+HPV were compared among the 3 groups over 8 weeks after the 2 doses of the HPV Vaccine was administrated (Fig 7).

Cells cultured in the presence of HPV Ag (1.65μg/ml) displayed a general increase of IFN-γ production after administration of the 1^st^ dose of vaccine. Four weeks later, HPV-Only group produced the highest level of IFN-γ (7.49±2.67pg/ml) while the Schisto-infected+HPV group produced the lowest (1.59±1.5pg/ml) however this difference was not significant. After administration of the 2^nd^ dose of the vaccine, there was an increase in IFN-γ levels observed in the 3 groups. At the 8^th^ week, Schisto-infected+HPV and Schisto/PZQ+HPV groups’ IFN-γ continued to rise slightly while a slight decline in IFN-γ the HPV-only group was observed. However, this was not statistically significant when comparison among the three groups was done.

SEA and SWAP stimulation produced relatively low and constant IFN-γ levels throughout the 8 weeks. However the HPV-Only group (Week 4) saw relatively high IFN-γ levels when the PBMCs were stimulated with SEA, this could be due to one extreme value from one animal of the group (38.91pg/ml). The difference among the three groups was not statistically significant.

Non-specific stimulation with CONA (20μg/ml) also saw a general increase of IFN-γ levels after administration of the 1^st^ dose of vaccine. IFN-γ was produced in high variable levels in the 3 groups. (Fig 7)

## Discussion

Protection against several diseases is highly dependent on the magnitude and quality of antibodies produced after vaccine administration. The HPV 16/18 bivalent vaccine induces high levels of protective antibody concentrations, both systemic and mucosal, in vaccinated individuals, [36]. Circulating systemic anti-HPV antibodies formed after vaccination contribute to the antibodies present in the reproductive tract which result in protection against infection of the keratinocytes [37,38].

In this study, we purposed to determine probable effects a chronic *Schistosoma mansoni* infection has on the levels of HPV specific IgG antibodies. During the study, substantial levels of HPV specific IgG antibodies were detected in the serum obtained from the 3 groups of animals that received 0.5ml of *Ceravix* vaccine. There was a considerable increase in HPV specific IgG antibody levels after the first dose of the vaccine was administered, with the HPV-Only group having the highest IgG antibody titers compared to the Schisto-infected+HPV and Schisto/PZQ+HPV group, throughout the 8 weeks. This increase in HPV specific IgG levels in serum after vaccination have been observed in several human clinical trial studies [28,36,39]

In this study, a delayed increase in HPV-specific IgG levels was observed in the group that underwent anti-helminthic treatment prior to vaccination (Schisto/PZQ+HPV group), as the highest antibody titers were observed 6 weeks after the 1^st^ vaccine dose (2 weeks after the 2^nd^ dose) as opposed to the HPV-only group which showed a relatively high level of HPV specific IgG antibodies 4 weeks after the 1^st^ vaccination and these levels rose further after the 2^nd^ vaccination. The HPV specific IgG level titers were slightly higher in the group that underwent anti-helminth treatment compared to the group with the chronic *S*. *mansoni* infection. This finding is in accordance with a study that investigated the effect anti-helmithic treatment has on the efficacy of the influenza and Tetanus toxoid vaccine [21,40].

The study provided an indication that the study subjects with chronic schistosomiasis were capable of responding to the vaccines and produced HPV specific IgG antibodies, however, after the 6^th^ week a decrease in IgG levels was observed. This reduced humoral response has also been observed in helminth infected subjects administered with a number of vaccines [22,40–42].

This rapid increase in HPV specific antibodies titers after the 1^st^ dose would provide a strong first line of defense against HPV if the individual gets infected. However, according to this study’s findings, an *S*. *mansoni* infection may result in a reduction in HPV specific IgG antibodies after the 1^st^ vaccine is administered and these levels may not rise even after the 2^nd^ booster is given. Therefore protection against HPV may not be maintained for a long period, hence putting the individual at risk of HPV infection over time. While individuals that have previously undergone anti-helminthic treatment (such as PZQ) may experience a slight delay in the increase of HPV specific IgG antibody titer. However, elimination of the *S*. *mansoni* appears to result in improved humoral response compared to what could occur if the helminth infection remains chronic and untreated.

Successful vaccinations should elicit effective lymphocyte responses such as proliferation. Previous studies have shown that vaccination with the HPV vaccine results in substantial levels of T lymphocyte proliferation [14,43–46].

In the current study, substantial cellular proliferation occurred upon specific and non-specific stimulation, with lower levels of proliferation observed in the PBMCs cultured in media alone (MED). PBMCs collected from Schisto/PZQ+HPV and HPV-Only groups showed higher Proliferating CFSE^low^ cells in response to the HPV Ag, especially 4 weeks after the 1^st^ vaccination. While the Schisto-infected+HPV group only showed high proliferation 6 weeks after the 1^st^ vaccination (2weeks after the booster). This increase in Proliferating CFSE^low^ cells are due to the cells, especially the Lymphocytes, expanding and differentiating into effector cells as a reaction to antigen recognition, resulting into rapid cytokine production [47,48]. The proliferation capacity of PBMCs from the Schisto/PZQ+HPV and HPV-Only groups decreased at the 6^th^ week and rose slightly at the 8^th^ week, while the Schisto-infected+HPV group proliferation capacity continued to rise at the 8^th^ week. The decline in proliferating CFSE^low^ cells could be an indication of the cells entering homeostasis decline prior to the formation of memory cells [49]. This sudden rise and decline in proliferation capacity is similar to what was observed in other vaccine studies [50]. High proliferating CFSE^low^ cells were observed in Schisto-infected+HPV and Schisto/PZQ+HPV during stimulation with SEA and SWAP. This is expected as helminth infections would stimulate the production of protective cytokines, such as IL-4, which are responsible for the activation and expansion of the Th2 cells [49].

The data from this study implies that individuals with a chronic *S*. *mansoni* infection may require 2 doses of the HPV bivalent vaccine to enable their lymphocytes to undergo effective proliferation.

HPV vaccination has been shown to induce increased T-cell proliferation which result in increased cytokine production which is important in the stimulation and maintenance of the humoral responses which are required for effective HPV Vaccine response[43] as well as viral clearance [44].

This study aimed to determine whether schistosomiasis triggers a Th2 biased profile caused by the shift from Th1 to Th2 that it typical of helminth infections, as well as to determine if elimination the *S*. *mansoni* infection would restore the Th1-Th2 balance. During the study, ample amounts of IL-4 and IFN-γ cytokines were released into the culture supernatants upon specific and non-specific stimulation of the PBMCs collected from the 3 groups during the 8 weeks. As expected, it was observed that prior to vaccination higher amounts of IL-4 cytokines were produced in the subjects that had chronic schistosomiasis (Schisto-infected+HPV) and those that were previously exposed to the infection (Schisto/PZQ+HPV) while the control group (HPV-only) had slightly higher levels of IFN-γ than IL-4 prior to vaccination. Higher levels of IL-4 in these 2 groups is typical as this cytokine is involved in production of IgE which play a role in eosinophil-mediated defense against helminths [48,49]. Higher levels of IFN-γ than IL-4 levels were maintained in the HPV-only group throughout the 8 weeks. Surprisingly, stimulation with HPV Ag resulted in production of higher levels of IFN-γ than IL-4 in PBMCs collected from the Schisto-infected+HPV group 4 weeks and 8 weeks after vaccination, while the Schisto/PZQ+HPV group has a strong Th2 response indicated by very high levels of IL-4 cytokines produced 4 weeks after the 1^st^ vaccination. This high Th1 response discerned in this study is quite different from another study that showed higher levels of IL-4 and significantly lower IFN-γ levels in *S*. *mansoni* infected mice administered with the HIV-1C vaccine [27]. In the same study, following a PZQ treatment for the elimination of parasites, resulted in continued high levels of Th2 cytokines following SEA stimulation, while stimulation with CONA resulted in an increase in IFN-γ levels and lower IL-4 levels compared to the infected subjects, thus concluding that PZQ treatment only partially restored the Th1 bias.

A stronger Th1 response after the 2^nd^ vaccination is similar to the lack of bias seen in a study that aimed to investigate the effect of *Schistosoma mansoni* infection on the TB vaccine, MVA85A candidate vaccine [20]. Investigations involving individuals vaccinated with HPV L1 VLPs have shown strong Th1 and Th2 response *in vitro* indicated by high levels of IFN-γ, IL-5 and IL-10, especially in individuals with strong antibody titers. It was reported that these strong antibody titres have a role in increasing T cell responses [43,44]. The high levels of IFN-γ observed would play a role in stimulating the B cells to produce IgG [49] which is important for effective HPV vaccine responses [51]. There is also evidence that SEA from the eggs released during a *S*. *mansoni* infection also stimulated the production of proinflammatory cytokines [52]. The findings of this study indicates that a chronic *S*. *mansoni* infection or previous exposure to schistosomiasis does not down-regulate Th1 cellular responses to HPV Vaccine.

The research had an important limitation in which the sample size of the baboons was narrowed to 10 (n = 3, 4, 3) due to the high maintenance costs for each animal. This small sample size could have contributed to the inconclusive cellular responses results. However from the data, there is no indication that Schistosomiasis reduces the HPV vaccine induced cellular responses. The second limitation is that, even though the baboons are physiologically, anatomically and genetically similar and disease progression and immune response to the disease are related, baboons are not humans, hence there will be differences.

We can conclude that the *S*. *mansoni* infection did interfere with the production of systemic HPV specific IgG antibodies, as the resulting humoral response was not as strong as it could have been if the subject did not have the infection or had undergone antihelminthic treatment prior to vaccination. There is also an indication that anti-helminthic treatment with PZQ prior to vaccination may be beneficial as it could improve humoral responses. This study implies that individuals with a chronic *S*. *mansoni* infection may require 2 doses of the HPV bivalent vaccine to enable their lymphocytes to undergo effective proliferation. The data also indicates that Schistosomiasis does not interfere with the cellular immunogenicity elicited by the HPV Vaccine. Policies for helminth treatment prior to HPV vaccination may be required so as to provide an efficient vaccine induced response.

## Supporting information

S1 Fig Gating strategy used for flow cytometry analysis of proliferating PBMCs. (DOCX)

S1 Table CFSE profiles for PBMCs. (DOCX)

S2 Table Katokatz data. (XLSX)

S3 Table Worm perfusion data. (XLSX)

S4 Table IgG ELISA data. (XLSX)

S5 Table Median Fluorescence Intensity (MFI) and Robust SD (rSD) for proliferating cells. (DOCX)

S6 Table IL-4 concentration data. (XLSX)

S7 Table IFN-γ concentration data. (XLSX)
