# Supplementary material for: An investigation into the role of chronic Schistosoma mansoni infection on Human Papillomavirus (HPV) vaccine induced protective responses
Source: PLoS Negl Trop Dis. 2019 Aug 26;13(8):e0007704. doi: 10.1371/journal.pntd.0007704 (PMC6730949; doi:10.1371/journal.pntd.0007704)
Supplement: S1 Fig — (DOCX) [file pntd.0007704.s001.docx]

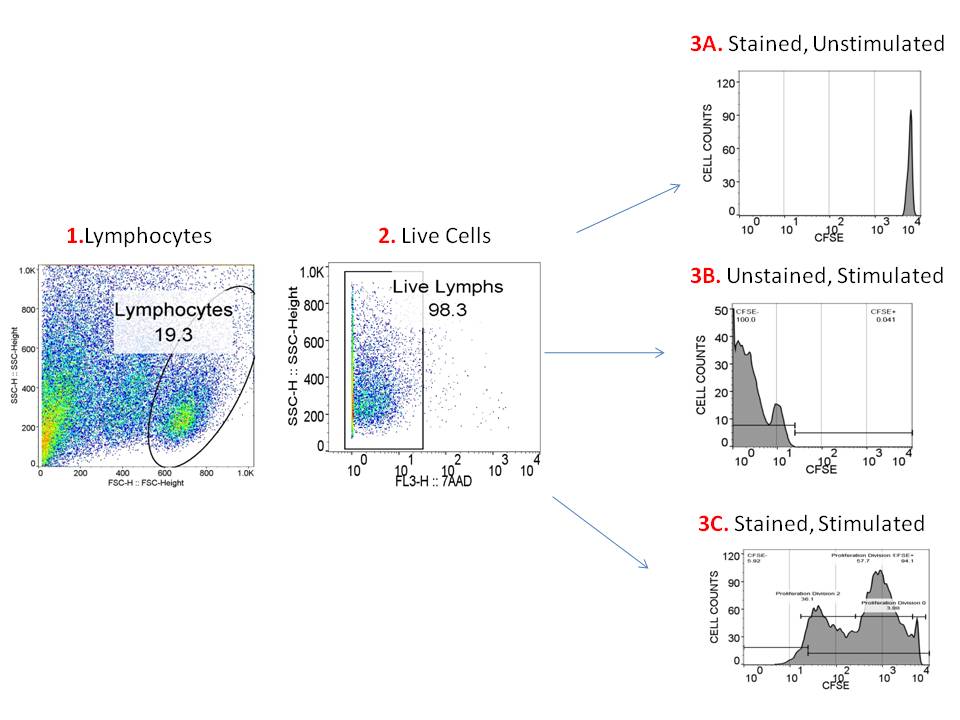


**S1 Fig. 1:** **Gating strategy used for flow cytometry analysis of Proliferating PBMCs.** The PBMCs were stimulated with appropriate stimulant, except for the negative control which was left unstimulated. The cells were plotted as forward Scatter (FSC) vs. Side Scatter (SSC) and the lymphocytes were gated (**Plot1**). The live cells were gated (**Plot 2**). The cells were then ploted as Histograms as CFSE (FITC) Vs. cell counts. The samples that were stained with CFSE and unstimulated provided the starting population (**Plot 3A**), the samples that were unstained and stimulated with ConA provided the autoflourescence of proliferating cells (**Plot3B**). Samples that were stained with CFSE and stimulated with the appropriate stimulant (ConA, HPV Ag, SEA or SWAP)(**Plot 3C**). The data acquired was then analysed using FlowJo Version 10 (FlowJo LLC, USA).
