# Supplementary material for: An investigation into the role of chronic Schistosoma mansoni infection on Human Papillomavirus (HPV) vaccine induced protective responses
Source: PLoS Negl Trop Dis. 2019 Aug 26;13(8):e0007704. doi: 10.1371/journal.pntd.0007704 (PMC6730949; doi:10.1371/journal.pntd.0007704)
Supplement: S1 Table — (DOCX) [file pntd.0007704.s002.docx]

**S1 Table 1.1:** **CFSE profile of PBMCs after 72hours in culture with HPV Ag**.

These CFSE profiles indicate the proliferation capacity of PBMCs collected from the 3 groups (Schisto-infected+HPV, Schisto/PZQ+HPV , HPV-only) during the 8 weeks after vaccination to respond in vitro to HPV Ag (20µl). The Non-dividing cells (light blue histogram) have the highest CFSE intensity, and the unlabeled stimulated cells (pink histogram) have the lowest CSFE intensity, Cells stimulated with HPV Ag (Orange histogram) had different number of peaks with different CFSE intensities. *missing data

| HPV Ag | | | | | | | | | | |
| --- | --- | --- | --- | --- | --- | --- | --- | --- | --- | --- |
| WEEKS | **Schisto-infected+HPV** | | | **Schisto/PZQ+HPV vaccine** | | | | **HPV- only (Control)** | | |
|  | **PAN3836** | **PAN3850** | **PAN4139** | **PAN3686** | **PAN3834** | **PAN3840** | **PAN3843** | **PAN3665** | **PAN3957** | **PAN3958** |
| 0 | 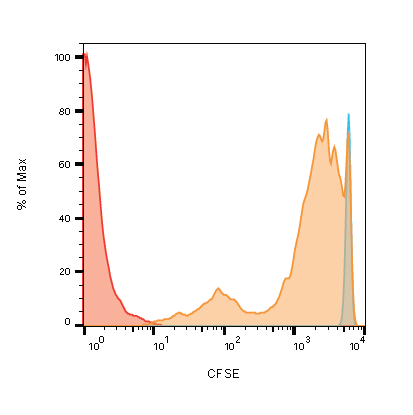 | 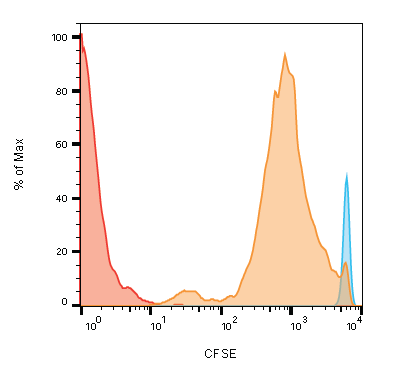 | 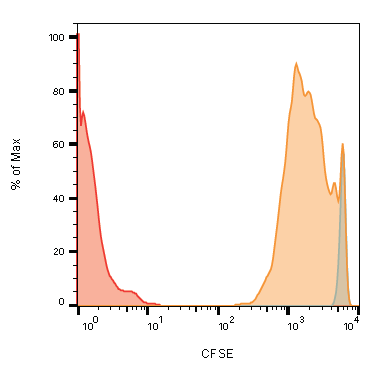 | 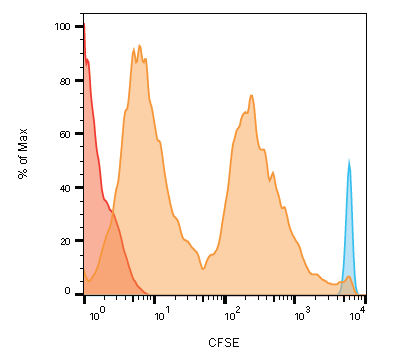 | 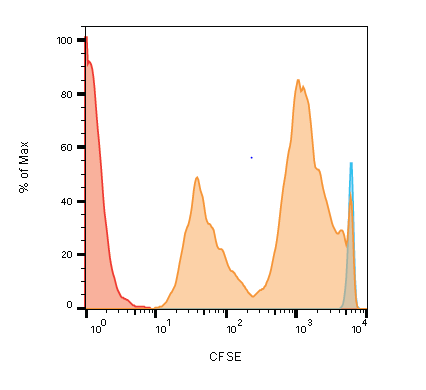 | 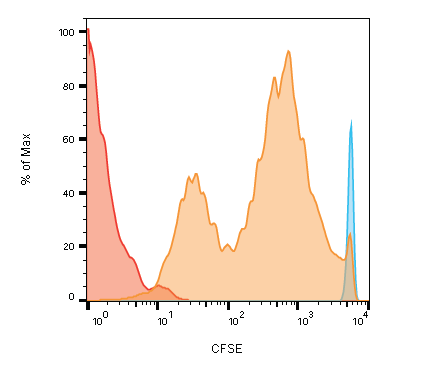 | 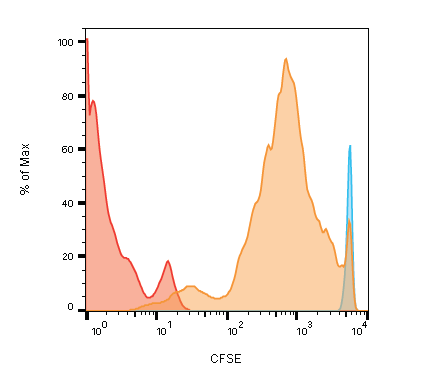 | 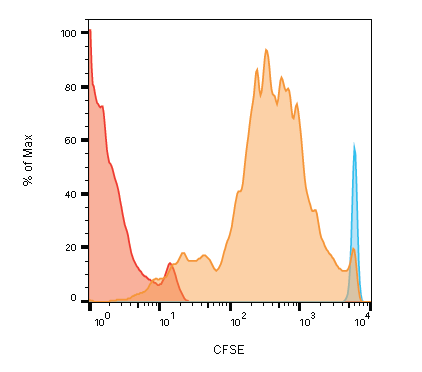 | 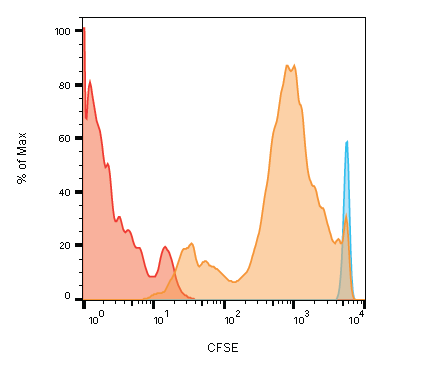 | 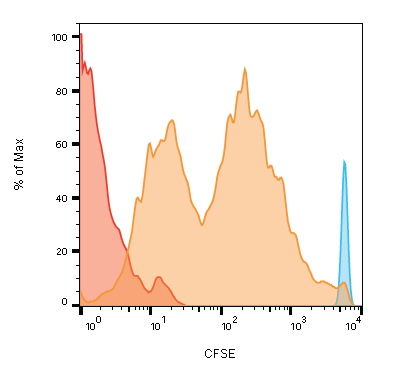 |
| 4 | 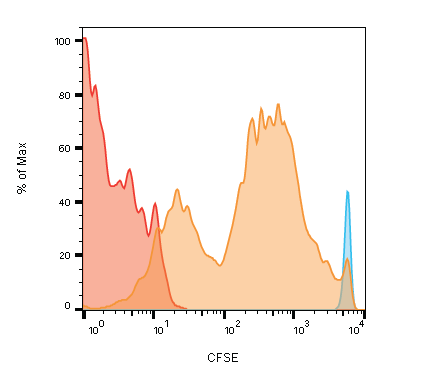 | 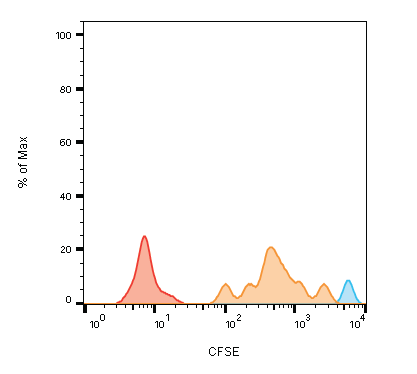 | 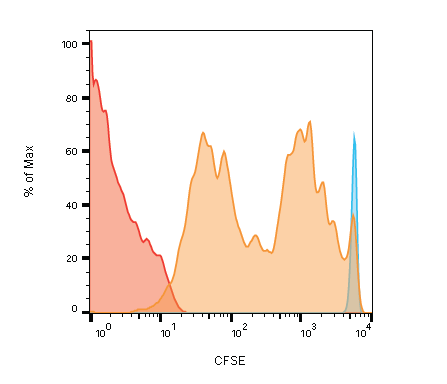 | 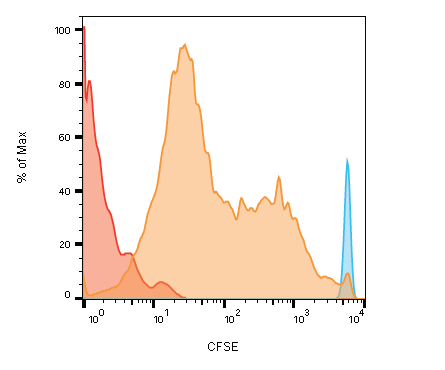 | 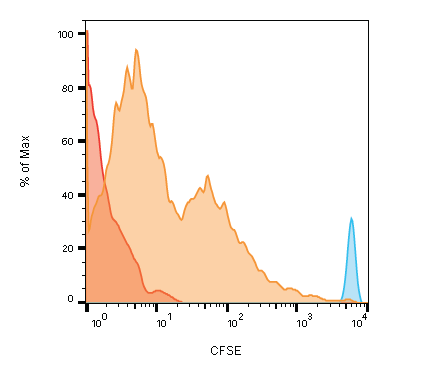 | 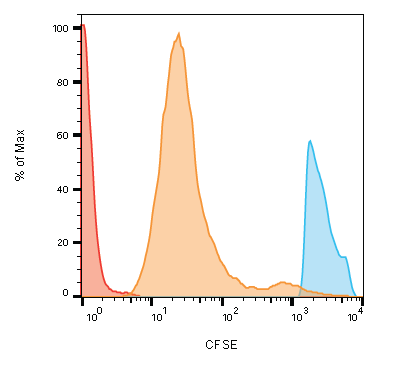 | 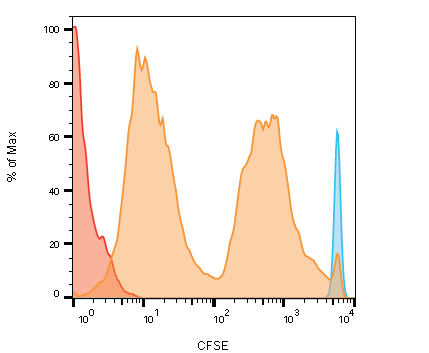 | 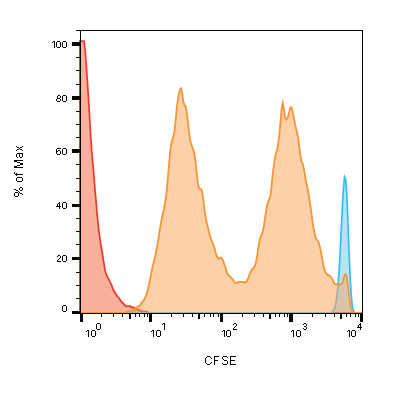 | 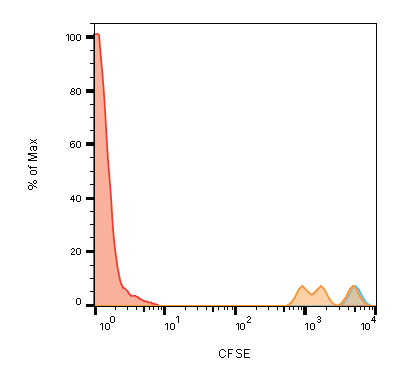 | 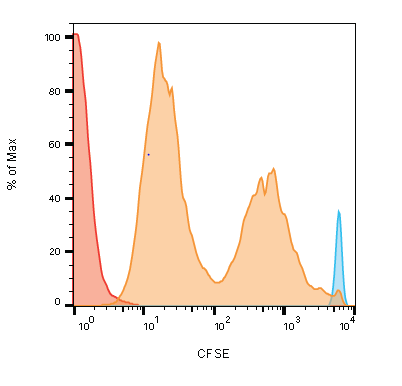 |
| 6 | 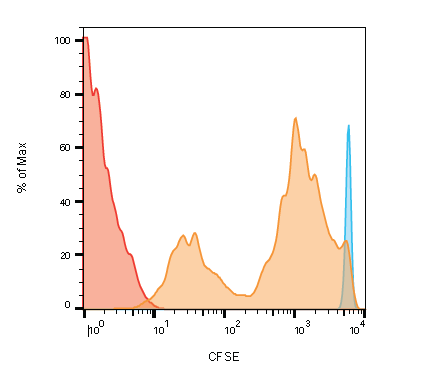 | 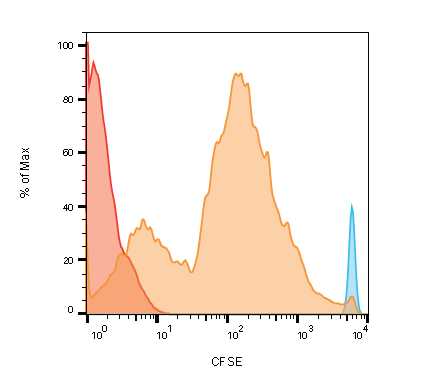 | 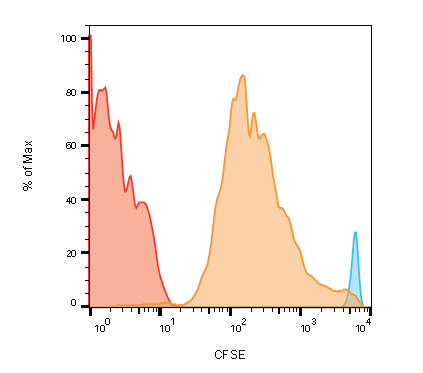 | 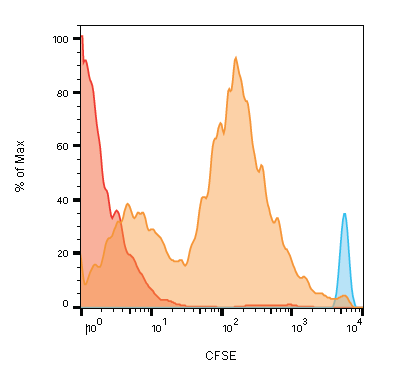 | 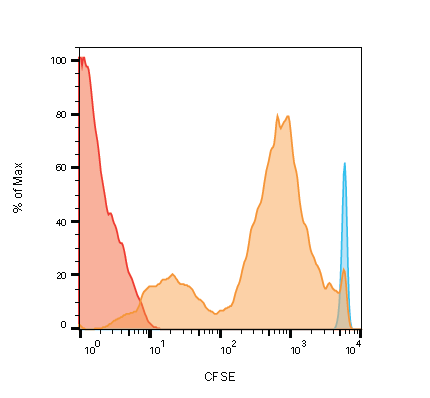 | * | 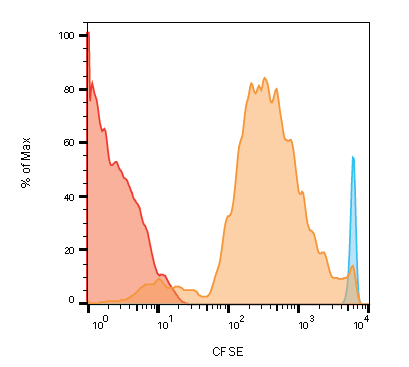 | * | 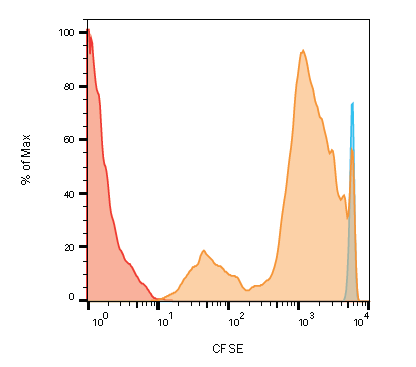 | 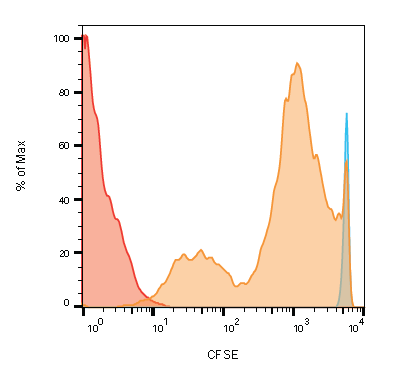 |
| 8 | 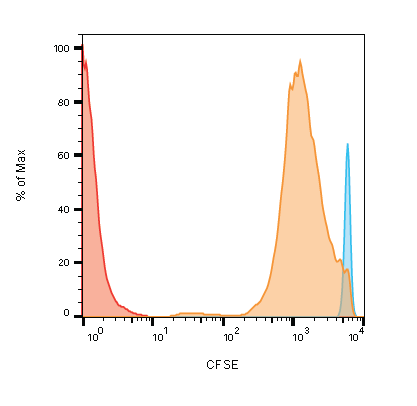 | 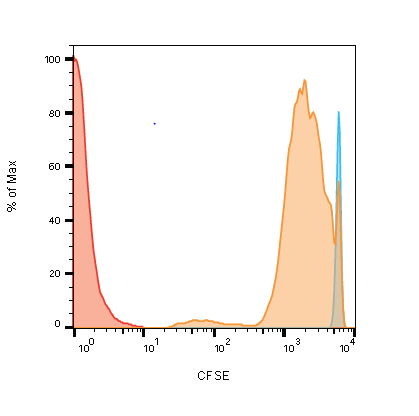 | 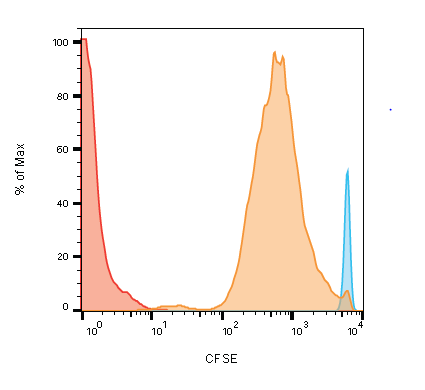 | * | 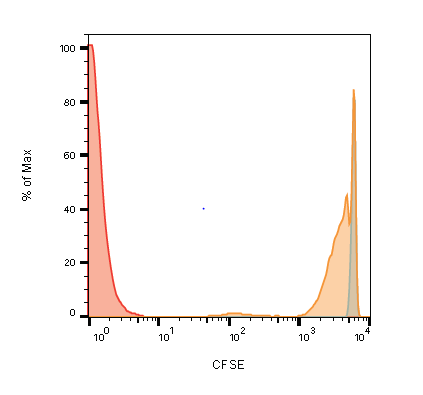 | 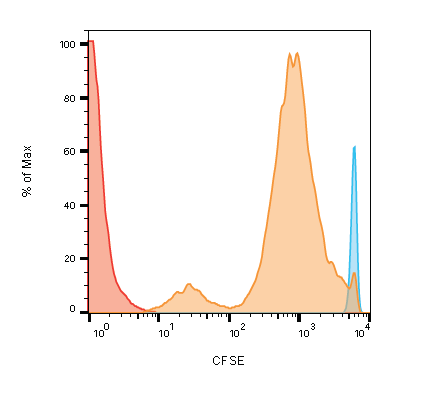 | 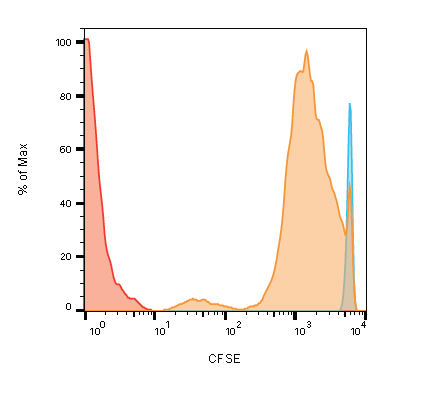 | 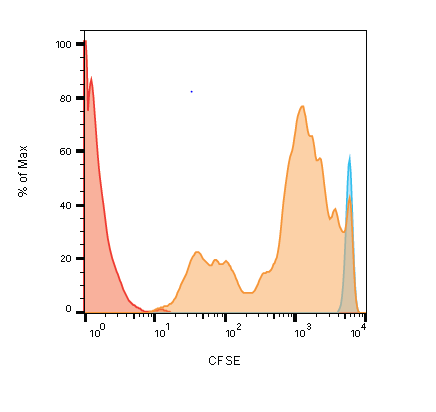 | 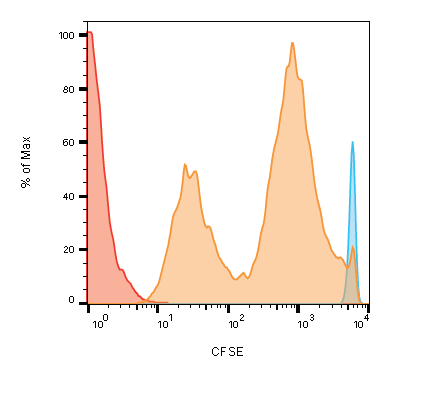 | 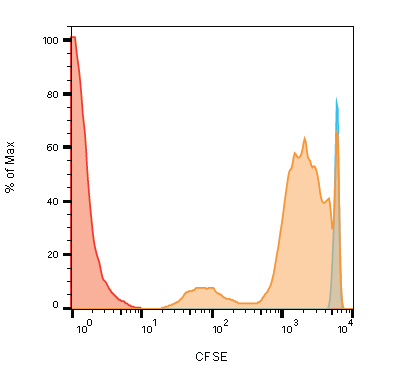 |

**S1 Table 1.2: CFSE profile of PBMCs after 72hours in culture with SEA.**

These CFSE profiles indicate the proliferation capacity of PBMCs collected from the 3 groups (Schisto-infected+HPV, Schisto/PZQ+HPV, HPV-only) during the 8 weeks after vaccination to respond in vitro to SEA (20µl). The Non-dividing cells (light blue histogram) have the highest CFSE intensity, and the unlabeled stimulated cells (pink histogram) have the lowest CSFE intensity, Cells stimulated with SEA (grey histogram) had different number of peaks with different CFSE intensities. *missing data

| SEA | | | | | | | | | | |
| --- | --- | --- | --- | --- | --- | --- | --- | --- | --- | --- |
|  | **Schisto-infected+HPV** | | | **Schisto/PZQ+HPV vaccine** | | | | **HPV-only (Control)** | | |
|  | **PAN3836** | **PAN3850** | **PAN4139** | **PAN3686** | **PAN3834** | **PAN3840** | **PAN3843** | **PAN3665** | **PAN3957** | **PAN3958** |
| 0 | 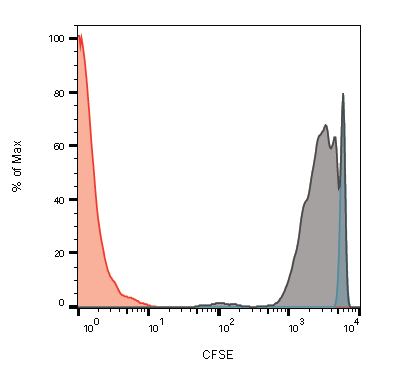 | 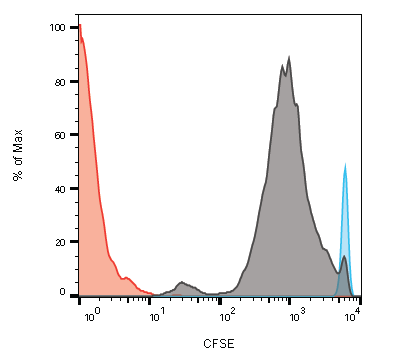 | 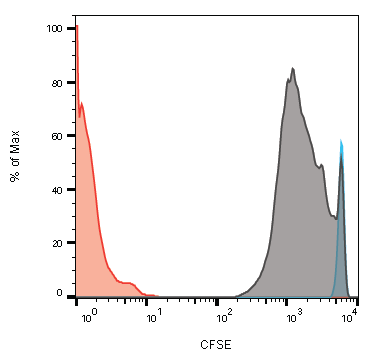 | 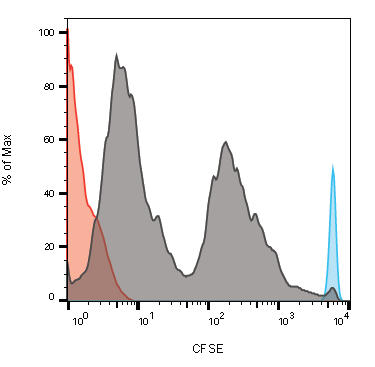 | 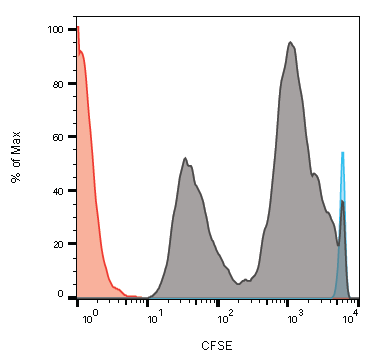 | 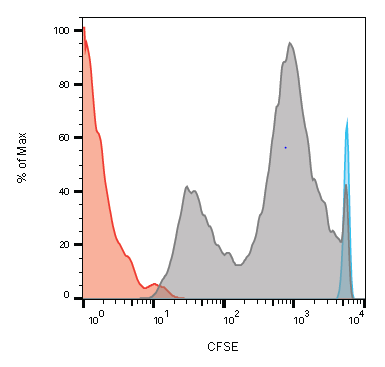 | 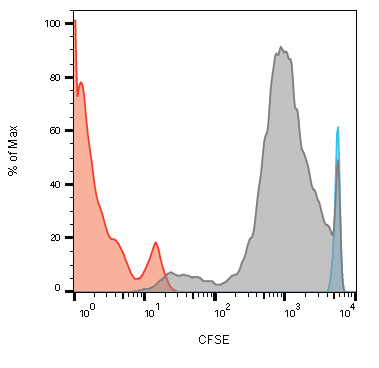 | 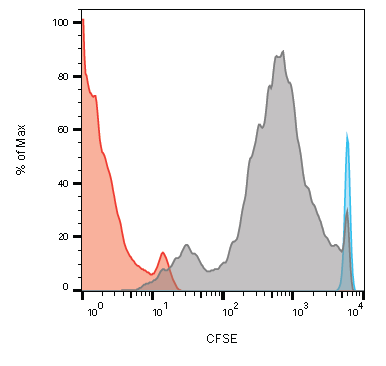 | 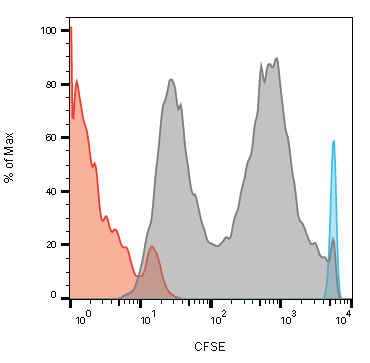 | 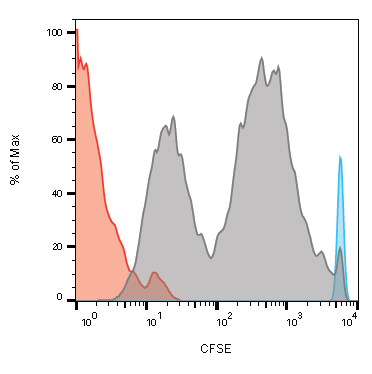 |
| 4 | 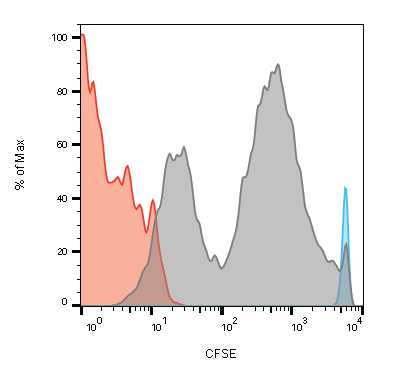 | 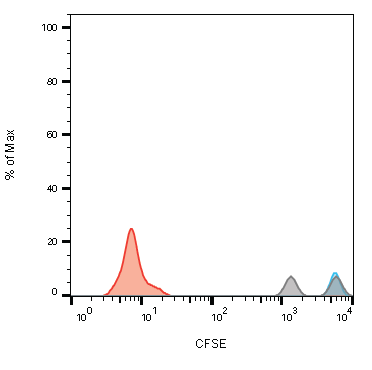 | 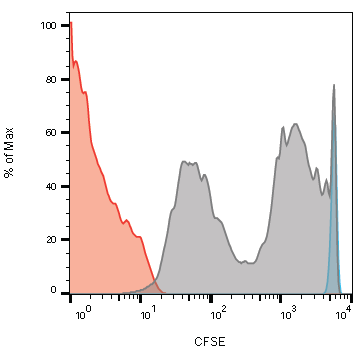 | 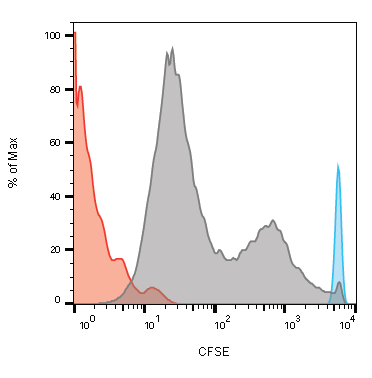 | 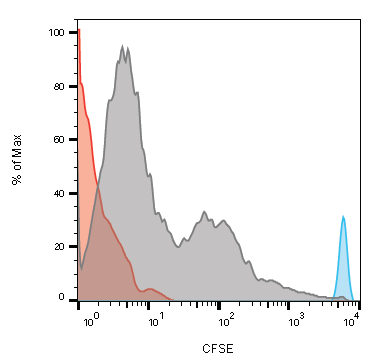 | 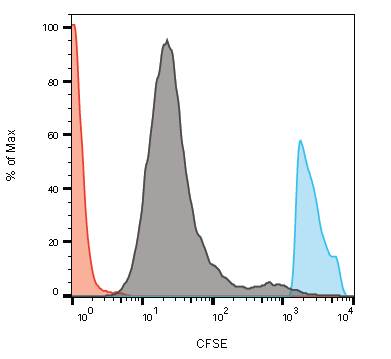 | 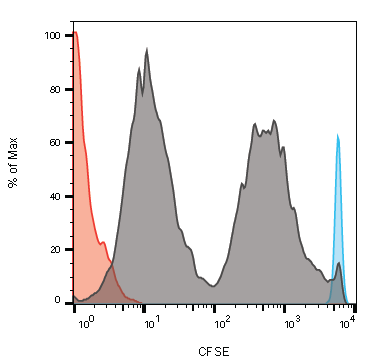 | 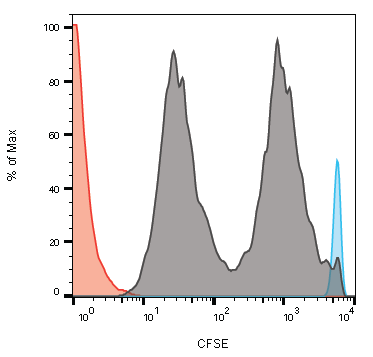 | 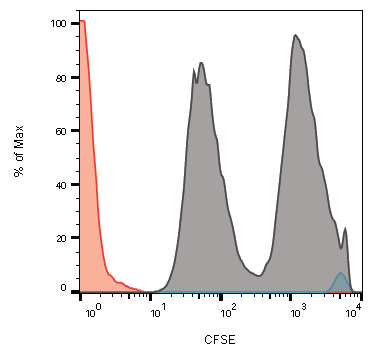 | 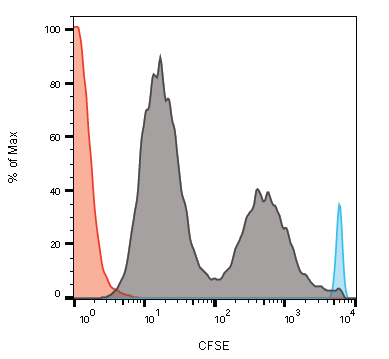 |
| 6 | 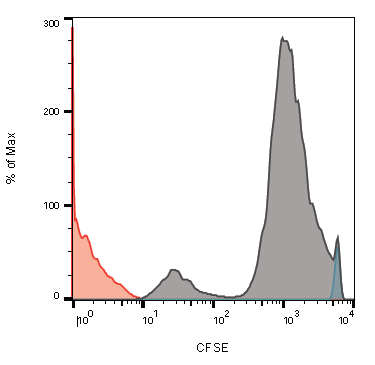 | 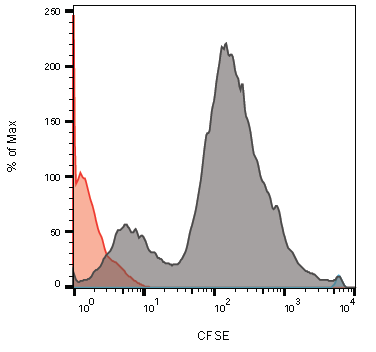 | 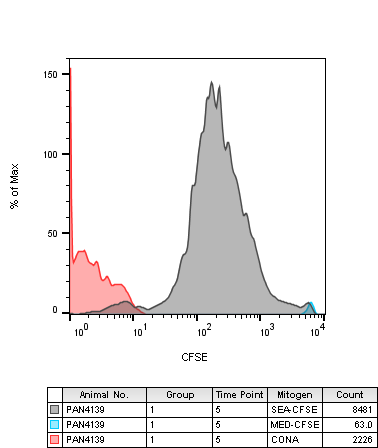 | 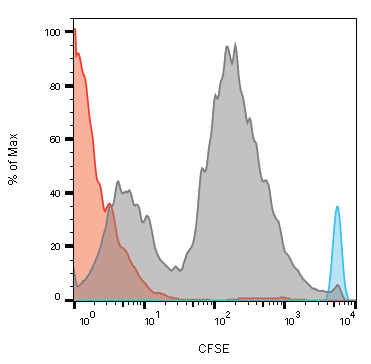 | 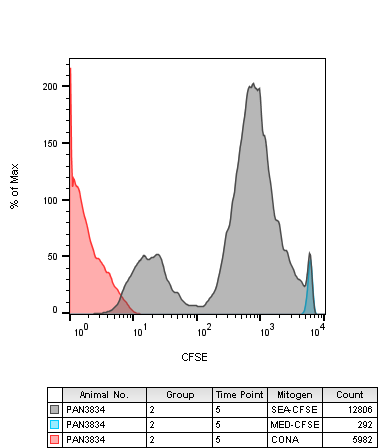 | * | 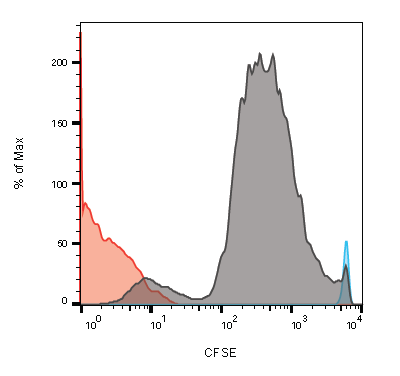 | * | 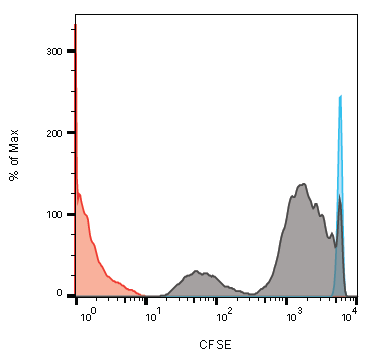 | 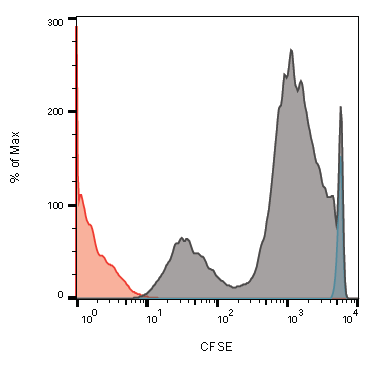 |
| 8 | 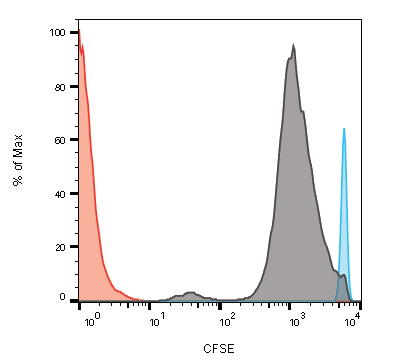 | 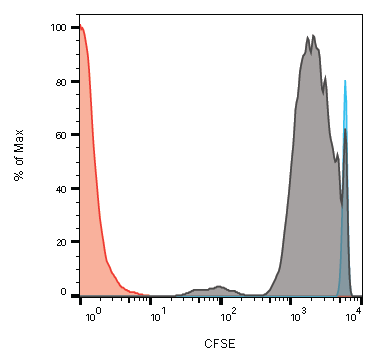 | 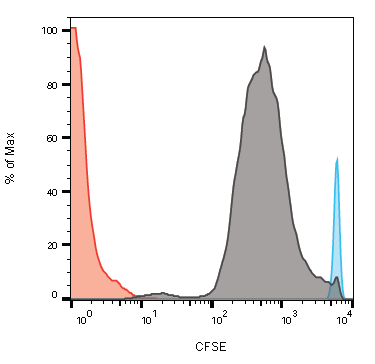 | * | 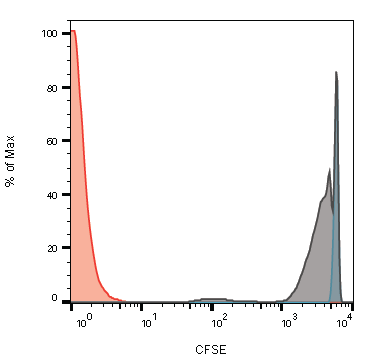 | 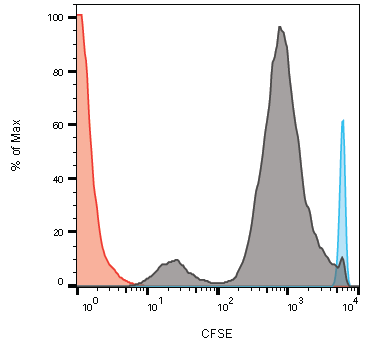 | 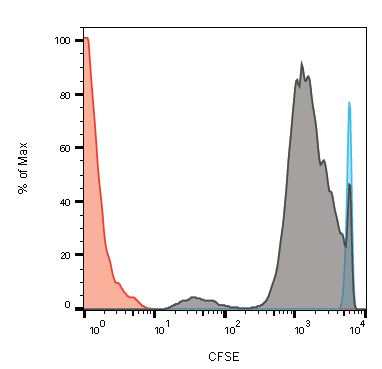 | 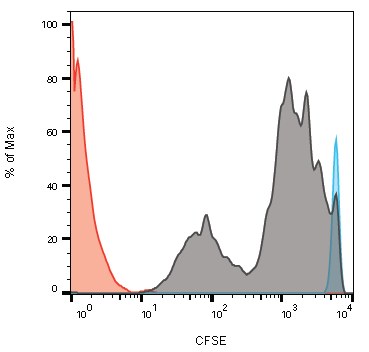 | 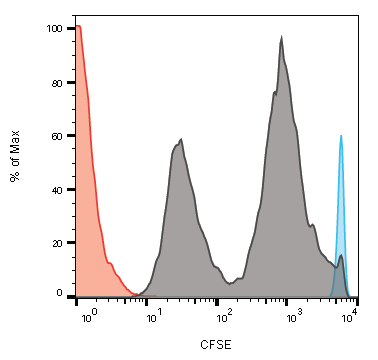 | 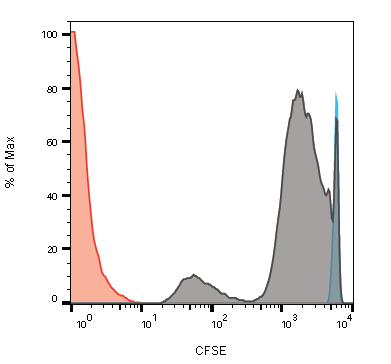 |

**S1 Table 1.3: CFSE profile of PBMCs after 72hours in culture with SWAP**.

These CFSE profiles indicate the proliferation capacity of PBMCs collected from the 3 groups (Schisto-infected+HPV, Schisto/PZQ+HPV, HPV-only during the 8 weeks after vaccination to respond in vitro to SWAP (20µl). The Non-dividing cells (light blue histogram) have the highest CFSE intensity, and the unlabeled stimulated cells (pink histogram) have the lowest CSFE intensity, Cells stimulated with SWAP (Brown histogram) had different number of peaks with different CFSE intensities. *missing data

| SWAP | | | | | | | | | | |
| --- | --- | --- | --- | --- | --- | --- | --- | --- | --- | --- |
|  | **Schisto-infected+HPV** | | | **Schisto/PZQ+HPV** | | | | **HPV-only (Control)** | | |
|  | **PAN3836** | **PAN3850** | **PAN4139** | **PAN3686** | **PAN3834** | **PAN3840** | **PAN3843** | **PAN3665** | **PAN3957** | **PAN3958** |
| 0 | 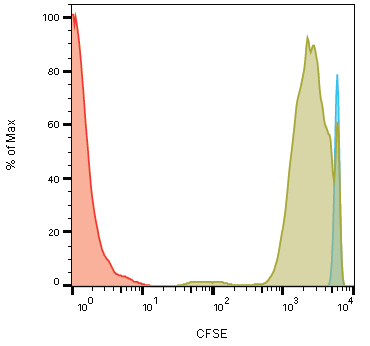 | 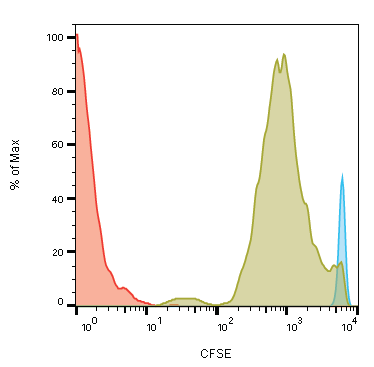 | 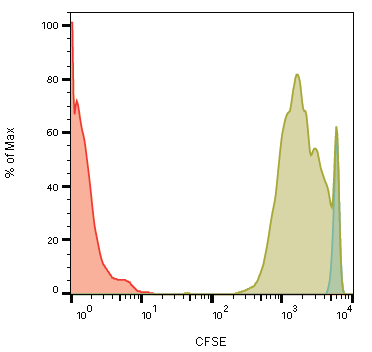 | 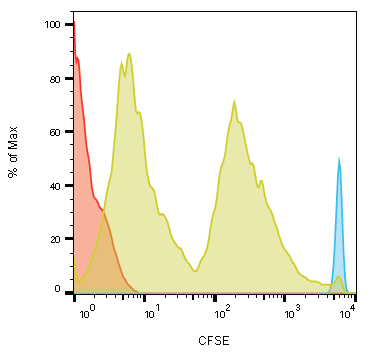 | 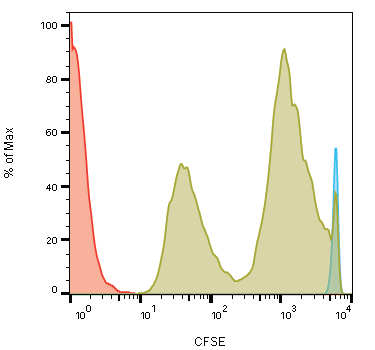 | 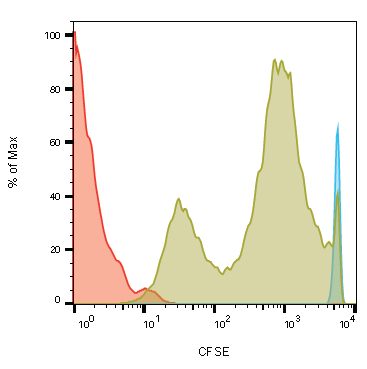 | 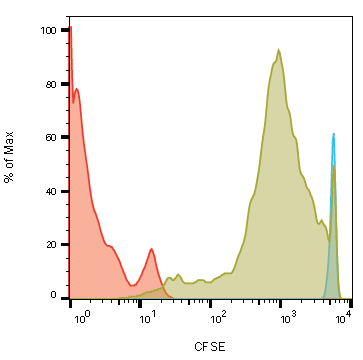 | 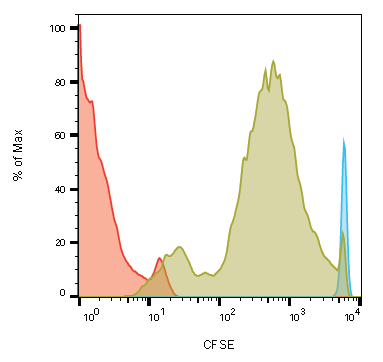 | 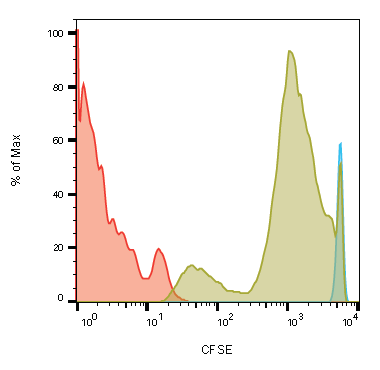 | 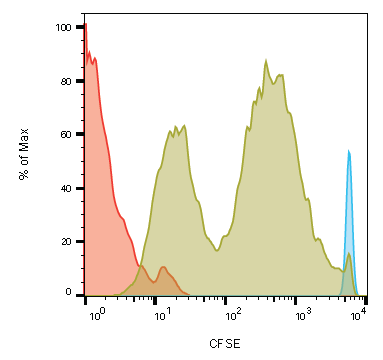 |
| 4 | 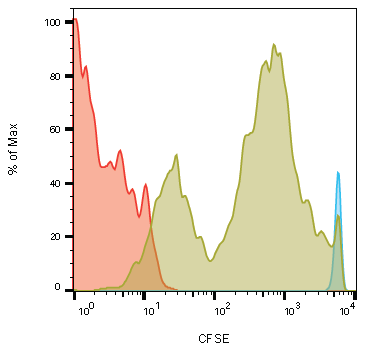 | 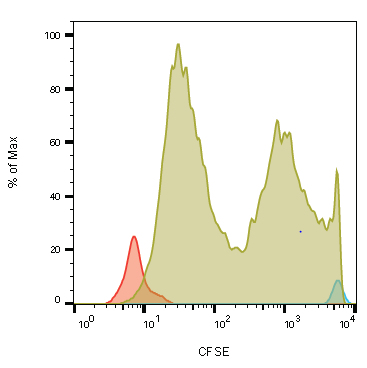 | 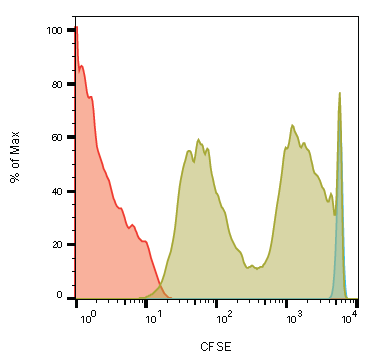 | 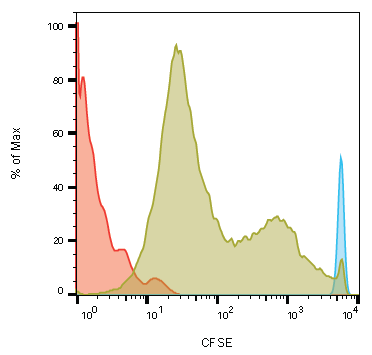 | 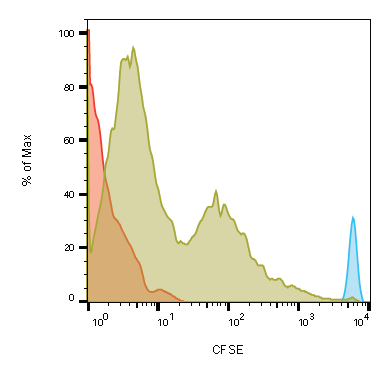 | 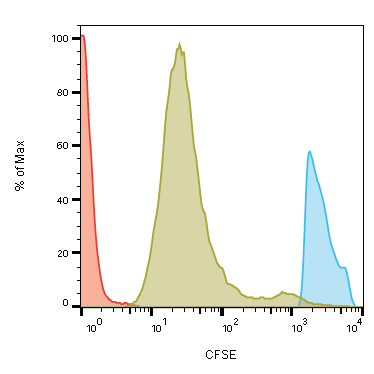 | 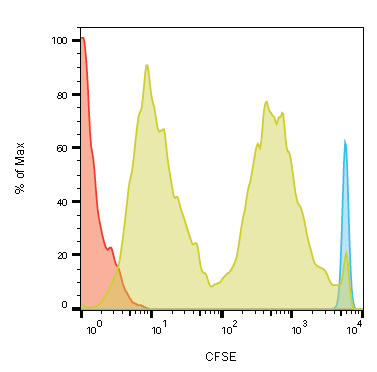 | 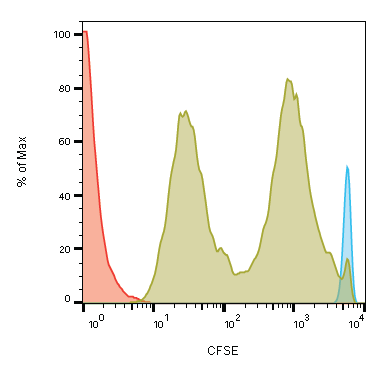 | 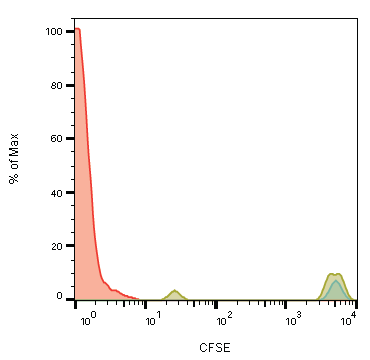 | 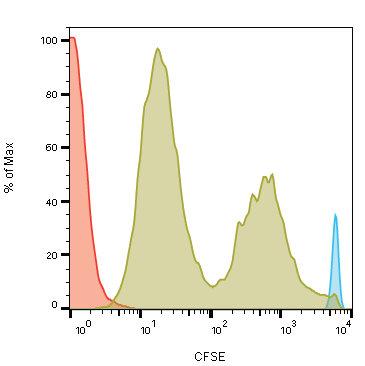 |
| 6 | 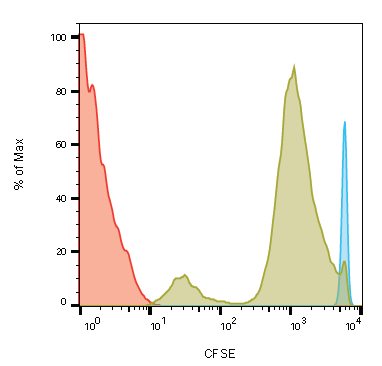 | 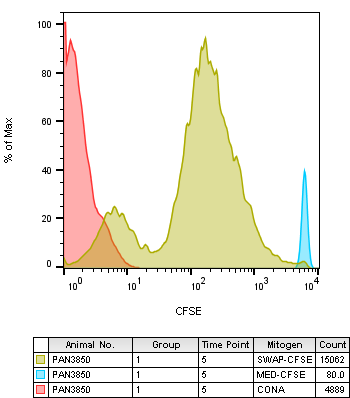 | 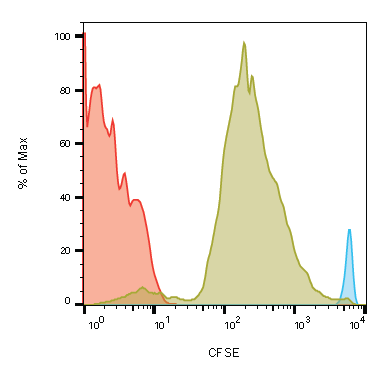 | 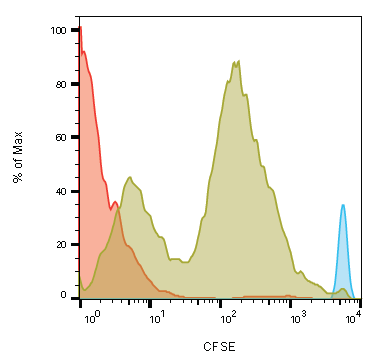 | 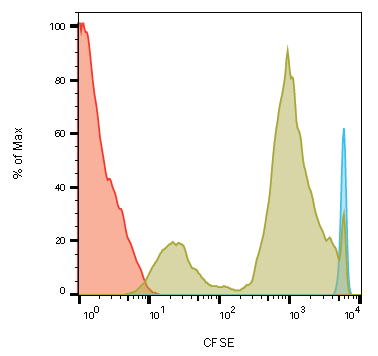 | * | 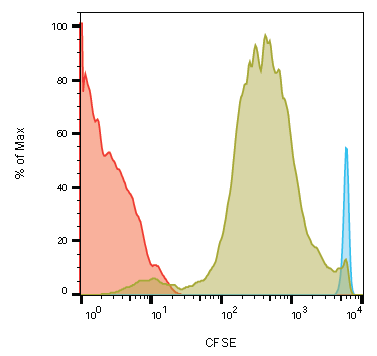 | * |  |  |
| 8 |  |  |  | * |  |  |  |  |  |  |

**S1 Table 1.4: CFSE profile of PBMCs after 72hours in culture with CONA**.

These CFSE profiles indicate the proliferation capacity of PBMCs collected from the 3 groups (Schisto-infected+HPV, Schisto/PZQ+HPV, HPV-only) during the 8 weeks after vaccination to respond in vitro to CONA (20µl). The Non-dividing cells (light blue histogram) have the highest CFSE intensity, and the unlabeled stimulated cells (pink histogram) have the lowest CSFE intensity, Cells stimulated with CONA (Purple histogram) had different number of peaks with different CFSE intensities. *missing data

| CONA | | | | | | | | | | |
| --- | --- | --- | --- | --- | --- | --- | --- | --- | --- | --- |
|  | **Schisto+HPV** | | | **Schisto/PZQ+HPV** | | | | **HPV-only (Control)** | | |
|  | **PAN3836** | **PAN3850** | **PAN4139** | **PAN3686** | **PAN3834** | **PAN3840** | **PAN3843** | **PAN3665** | **PAN3957** | **PAN3958** |
| 0 |  |  |  |  |  |  |  |  |  |  |
| 4 |  |  |  |  |  |  |  |  |  |  |
| 6 |  |  |  |  |  | * |  | * |  |  |
| 8 |  |  |  | * |  |  |  |  |  |  |
