# Supplementary material for: An investigation into the role of chronic Schistosoma mansoni infection on Human Papillomavirus (HPV) vaccine induced protective responses
Source: PLoS Negl Trop Dis. 2019 Aug 26;13(8):e0007704. doi: 10.1371/journal.pntd.0007704 (PMC6730949; doi:10.1371/journal.pntd.0007704)
Supplement: S5 Table — (DOCX) [file pntd.0007704.s006.docx]

**S1 Table 2:** **Median Fluorescence Intensity (MFI) and Robust SD (rSD) for proliferating cells.** PBMCs were labelled with CFSE and stimulated in vitro with HPV Ag, SEA, SWAP and CONA for 72 hours. Cells were analysed using flowcytometry to determine the dividing (decreasing CFSE) PBMCs. Decrease of MFI is inversely proportional to the increase of cell number.

|  | **MED** | | | | | | | | | |
| --- | --- | --- | --- | --- | --- | --- | --- | --- | --- | --- |
|  | **Schisto-infected+HPV** | | | **Schisto/PZQ+HPV** | | | | **HPV-Only** | | |
|  | **PAN3836** | **PAN3850** | **PAN4139** | **PAN3686** | **PAN3834** | **PAN3840** | **PAN3843** | **PAN3665** | **PAN3957** | **PAN3958** |
| 0 | 5790±349 | 6016±306 | 5882±353 | 5829±328 | 5909±345 | 5523±333 | 5511±347 | 5882±276 | 5511±319 | 5687±287 |
| 4 | 5700±303 | 5573±0 | 5636±339 | 5661±310 | 5829±203 | 2334±1101 | 5674±353 | 5674±396 | 5071±0 | 5869±306 |
| 6 | 5764±310 | 5856±229 | 5909±379 | 5486±392 | 5764±302 |  | 5909±321 |  | 5700±304 | 5548±327 |
| 8 | 5816±343 | 5829±337 | 5949±304 |  | 5856±320 | 5909±348 | 5856±371 | 5790±378 | 5764±384 | 5843±374 |
|  | **HPV Ag** | | | | | | | | | |
|  | **Schisto-infected+HPV** | | | **Schisto/PZQ+HPV** | | | | **HPV-Only** | | |
|  | **PAN3836** | **PAN3850** | **PAN4139** | **PAN3686** | **PAN3834** | **PAN3840** | **PAN3843** | **PAN3665** | **PAN3957** | **PAN3958** |
| 0 | 1907±1666 | 800±644 | 1647±1117 | 178±275 | 876±1046 | 491±616 | 675±681 | 407±489 | 833±803 | 231±326 |
| 4 | 414±515 | 479±477 | 340±772 | 165±389 | 75.2±105 | 26.2±23.5 | 281±457 | 257±624 | 884±0 | 40±346 |
| 6 | 902±1076 | 167±233 | 201±259 | 209±240 | 625±679 |  | 364±441 |  | 1200±1223 | 933±1060 |
| 8 | 1266±838 | 1864±1118 | 598±464 |  | 3391±1237 | 781±622 | 1423±1034 | 1053±1196 | 571±696 | 1743±1287 |
|  | **SEA** | | | | | | | | | |
|  | **Schisto-infected+HPV** | | | **Schisto/PZQ+HPV** | | | | **HPV-Only** | | |
|  | **PAN3836** | **PAN3850** | **PAN4139** | **PAN3686** | **PAN3834** | **PAN3840** | **PAN3843** | **PAN3665** | **PAN3957** | **PAN3958** |
| 0 | 2672±1308 | 862±648 | 1430±1058 | 153±233 | 826±987 | 712±831 | 952±820 | 595±641 | 539±625 | 421±518 |
| 4 | 469±578 | 1313±0 | 703±1147 | 154±415 | 98.2±143 | 25±22.7 | 285±476 | 249±622 | 561±880 | 32.3±306 |
| 6 | 1102±784 | 181±226 | 207±228 | 219±242 | 672±649 |  | 426±431 |  | 1452±1432 | 1067±1163 |
| 8 | 1147±726 | 1920±1127 | 529±421 |  | 3338±1270 | 764±569 | 1420±1001 | 1132±1245 | 580±680 | 1692±1170 |
|  | **SWAP** | | | | | | | | | |
|  | **Schisto-infected+HPV** | | | **Schisto/PZQ+HPV** | | | | **HPV-Only** | | |
|  | **PAN3836** | **PAN3850** | **PAN4139** | **PAN3686** | **PAN3834** | **PAN3840** | **PAN3843** | **PAN3665** | **PAN3957** | **PAN3958** |
| 0 | 2267±1177 | 817±648 | 1677±1158 | 181±258 | 849±1007 | 712±810 | 910±873 | 532±553 | 1186±945 | 399±481 |
| 4 | 540±641 | 326±791 | 519±1081 | 117±446 | 89.9±127 | 28±243 | 336±495 | 368±639 | 26.6±0 | 37.7±346 |
| 6 | 1077±768 | 189±216 | 228±242 | 218±232 | 896±915 |  | 418±422 |  | 1655±1085 | 1034±1217 |
| 8 | 1200±791 | 1902±1132 | 535±408 |  | 3531±1211 | 791±616 | 1611±1069 | 1046±1281 | 591±687 | 1754±1148 |
|  | **CONA** | | | | | | | | | |
|  | **Schisto-infected+HPV** | | | **Schisto/PZQ+HPV** | | | | **HPV-Only** | | |
|  | **PAN3836** | **PAN3850** | **PAN4139** | **PAN3686** | **PAN3834** | **PAN3840** | **PAN3843** | **PAN3665** | **PAN3957** | **PAN3958** |
| 0 | 2864±1317 | 797±644 | 1607±1098 | 174±273 | 701±926 | 555±782 | 862±916 | 580±734 | 506±648 | 336±525 |
| 4 | 270±577 | 1582±1512 | 139±935 | 148±422 | 92.6±170 | 29.7±27.3 | 266±489 | 281±678 | 804±0 | 34.7±323 |
| 6 | 1301±1355 | 231±352 | 360±454 | 273±356 | 748±929 |  | 432±473 |  | 1568±1652 | 1053±1338 |
| 8 | 1389±1030 | 2021±1319 | 710±608 |  | 3148±1975 | 833±795 | 1719±1291 | 1224±1398 | 498±761 | 1928±1736 |
